# Genetic Engineering Approaches for the Microbial Production of Vanillin

**DOI:** 10.3390/biom14111413

**Published:** 2024-11-06

**Authors:** Luísa D. F. Santos, Sylvie Lautru, Jean-Luc Pernodet

**Affiliations:** CEA, CNRS, Institute for Integrative Biology of the Cell (I2BC), Université Paris-Saclay, 91198 Gif-sur-Yvette, France; luisa.ferreira-dos-santos@universite-paris-saclay.fr

**Keywords:** vanillin, bioconversion, metabolic engineering, pathway design, synthetic biology, microbial production, accessible raw material

## Abstract

Vanilla flavour is widely used in various industries and is the most broadly used flavouring agent in the food industry. The demand for this flavour is, therefore, extremely high, yet vanilla bean extracts can only meet about 1% of the overall demand. Vanillin, the main constituent of vanilla flavour, can easily be obtained through chemical synthesis. Nonetheless, consumer demands for natural products and environmentally friendly industrial processes drive the development of biotechnological approaches for its production. Some microorganisms can naturally produce vanillin when fed with various substrates, including eugenol, isoeugenol, and ferulic acid. The characterisation of the genes and enzymes involved in these bioconversion pathways, as well as progress in the understanding of vanillin biosynthesis in *Vanilla* orchids, allowed the development of genetic engineering and synthetic biology approaches to increase vanillin production in naturally vanillin-producing microorganisms, or to implement novel vanillin biosynthetic pathways in microbial chassis. This review summarises and discusses these genetic engineering and synthetic biology approaches for the microbial production of vanillin.

## 1. Introduction

Vanilla flavour is widely used in the food, beverage, cosmetics, pharmaceutical, and tobacco industries. Natural vanilla flavour comprises dozens of organic compounds extracted from cured pods of different species of vanilla orchids, namely *Vanilla planifolia*, *V. pompon,* and *V. tahitensis* [1] (and references therein). The chemical composition of vanilla extracts depends on the plant species, cultivation conditions, curing and extraction methods, and storage conditions. The main constituent of vanilla flavour is vanillin (4-hydroxy-3-methoxy benzaldehyde), whose concentration can reach up to 2% *w*/*w* in cured vanilla pods [2]. Pure vanillin is widely used to boost other sweet food flavours, break down acidity, or mask the bitterness of specific food components. Furthermore, vanillin may serve as a food preservative in several dairy products, and its use in bakery significantly reduces the addition of sugar [3].

The global vanillin market was about 37,000 tonnes in 2018 and is estimated to exceed 59,000 tonnes in 2025 [4]. *Vanilla* orchids, originally from Mexico, are currently cultivated around the tropics (in a total area of around 97,800 ha in 2018) [2]. However, vanilla beans can only meet about 1% of the annual vanillin market demand. Vanilla cultivation is labour-intensive, has a low yield of beans (around 75 kg/ha), and the extraction processes are laborious and time-consuming, making vanilla extracts very expensive. The chemical synthesis of vanillin, e.g., from guaiacol and lignin, is an economical alternative. However, the lack of regioselectivity of some synthetic steps can reduce the process efficiency and increase the downstream processing costs [2]. Moreover, chemical synthesis processes require the use of hazardous chemicals (e.g., oxidising agents in alkaline conditions) and have adverse environmental impacts. Nevertheless, chemically synthesised vanillin represents around 90% of the American market, and its price is about a hundred times lower than the one of natural extracts from *Vanilla* beans.

While, according to US food regulation, vanillin produced from a natural precursor by chemical synthesis is considered a “natural flavour” (FDA 101.22), by the European food regulation, the label “natural flavour” requires natural precursors and enzymatic or fermentative transformation processes (UE 1334/2000). “Natural vanillin flavour” has an intermediate price (roughly ten times more than chemically synthesised vanillin, but ten times less than natural vanilla extract), and its market share is around 9% of the global vanillin market. The increasing demand for environmentally friendly processes and for “natural flavours” has led to the development of biotechnological processes for the bioproduction of vanillin at the industrial level [2]. The demand for vanillin produced by biotechnological processes, also called bio-vanillin, has increased, so manufacturers are expanding their production capacity and developing new strains and production processes [5].

The biotechnological production of vanillin requires prior knowledge of the native biosynthetic pathway of vanillin in orchids and/or the de novo design of biosynthetic pathways using a retrobiosynthetic approach and expert knowledge of specific biochemical reactions [6,7,8,9]. In this context, several studies have been performed to identify and characterise the enzymes and genes involved in the biosynthesis of vanillin in *Vanilla* [7,10]. These studies will be described in detail in Section 2. 

Since the early 1990s, several microorganisms (hereafter defined as vanillin-producing microorganisms) were identified for their natural capacity to produce vanillin from eugenol, isoeugenol, ferulic acid, or lignin [11,12,13]. Over the past three decades, considerable progress has been made in the molecular and biochemical characterisation of the genes and enzymes involved in the conversion of these various precursors into vanillin, and the diverse pathways that can be used for bioconversion have been the subject of several reviews [11,12,13]. These reviews have focused mainly on pathway characterisation and gene identification in vanillin-producing microorganisms. However, few details on the genetic engineering approaches to improve vanillin biosynthesis have been provided.

After briefly describing the current knowledge of the genes and enzymes involved in vanillin biosynthesis in plants (Section 2) and in vanillin-producing microorganisms (Section 3), our review focuses on the major genetic engineering or synthetic biology approaches used to produce vanillin in microorganisms (Section 4). These approaches aim either at improving vanillin yields in vanillin-producing microorganisms and at expressing, in these microorganisms, genes that enable them to use economically viable, water-soluble, and nontoxic precursors, or at introducing bioconversion (mainly from phenylpropanoids) or de novo biosynthetic pathways (from simple precursors such as sugars or from wastes such as polyethylene terephthalate residues) in non-producing strains that can be easily manipulated genetically.

## 2. Vanillin Biosynthesis in *Vanilla*

Early studies have established that vanillin is present in green *Vanilla* beans as vanillin glucoside, which is hydrolysed during curing to form the active flavour compound [10,14,15]. To elucidate the biosynthetic route to vanillin glucoside, green *Vanilla* beans were fed with several [^14^C]-radiolabelled molecules, including L-phenylalanine, L-tyrosine, shikimate intermediates, or lignin precursors [10,16]. Based on these first experiments, vanillin biosynthesis in *Vanilla* was assumed to be synthesised by the phenylpropanoid route [17].

It is generally accepted that vanillin is mainly produced from L-phenylalanine. A phenylalanine ammonia-lyase (PAL) converts L-phenylalanine to trans-cinnamic acid, which is then converted into 4-coumaric acid by a cinnamate-4-hydroxylase (C4H) [18]. PAL enzymes may also exhibit tyrosine ammonia-lyase activity (TAL), forming 4-coumaric acid directly from tyrosine. Feeding experiments with [^14^C]-L-tyrosine or [^14^C]-L-phenylalanine confirmed the existence of both PAL and TAL activities in *Vanilla* and suggested that PAL activity is ten times stronger than TAL activity [15]. 

**Figure 1:**
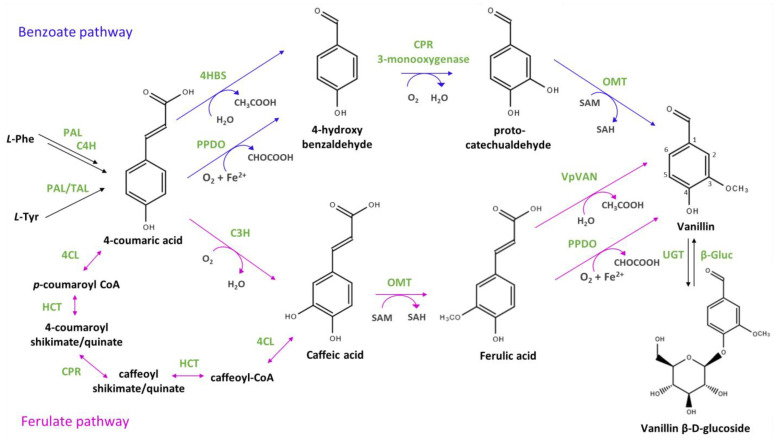
**Proposed vanillin biosynthetic pathways through 4-hydroxybenzandehyde and ferulic acid in *V. planifolia***. Based on Podstolski et al. [16], Gallage et al. [20], and Negishi and Negishi [21]. PAL: phenylalanine ammonia-lyase; C4H: cinnamate-4-hydroxylase; TAL: tyrosine ammonia-lyase; 4HBS: 4-hydroxybenzaldehyde synthetase; PPDO: phenylpropanoid 2,3-dioxygenase; CPR: cytochrome P450 reductase; OMT: *O*-methyltransferase; 4CL: 4-hydroxycinnamoyl-CoA ligase; HCT: hydroxycinnamoyl transferase; C3H: 4-coumaric acid 3-hydroxylase; VpVAN: vanillin synthase; UGT: UDP-glycosyltransferase; β-Gluc: β-glucosidase.

The conversion of 4-coumaric acid to vanillin requires four steps: (a) the shortening of the side chain by two carbons; (b) the introduction of the aldehyde function; (c) the introduction of the hydroxyl group at position 3; and (d) the O-methylation of this hydroxyl group (Figure 1). While O-methylation must occur after the 3-hydroxylation, the actual order of these reactions is still unresolved [17]. Some models propose that the aldehyde function is introduced during the chain-shortening reaction [2,11,19]. Some of the enzymes catalysing these reactions are still unidentified or poorly characterised in *Vanilla*, and their role in vanillin biosynthesis has been inferred based on studies of orthologous enzymes in other plants [10].

Based on the [^14^C]-radiolabelled precursor incorporation and the analysis of in vitro enzyme activities, two main routes have been proposed: the “benzoate pathway” and the “ferulate pathway” (Figure 1). The benzoate pathway involves the initial reduction of 4-coumaric acid to 4-hydroxybenzaldehyde, followed by hydroxylation and methylation. In contrast, the ferulate pathway proposes the hydroxylation and methylation of 4-coumaric acid prior to the side-chain reduction of ferulic acid.

The benzoate pathway was proposed by the Dixon group [16] who partially purified from *V. planifolia* tissue cultures an enzyme, designated as 4-hydroxybenzaldehyde synthetase (4HBS), catalysing in vitro the conversion of 4-coumaric acid into 4-hydroxybenzaldehyde. Conversion of 4-hydroxybenzaldehyde into vanillin was then proposed to proceed via hydroxylation at C3 followed by the methylation of the newly introduced hydroxyl group. On the other hand, in support of the ferulate pathway, Gallage et al. [20] identified in *V. planifolia* an enzyme, which they named vanillin synthase, encoded by the *VpVan* gene. When expressed in vitro, *Vp*VAN converted ferulic acid to vanillin, but showed no activity with 4-coumaric or caffeic acids. Later on, another side chain shortening enzyme, named phenylpropanoid 2,3-dioxygenase, was partially purified from *V. planifolia* and reported converting ferulic acid into vanillin, but also coumaric acid into 4-hydroxybenzaldehyde and caffeic acid into protocatechualdehyde in the presence of Fe^2+^ and thiol reagents [21]. Notably, in the presence of glutathione, ferulic acid and 4-coumaric acid were converted at similar rates into vanillin and 4-hydroxybenzaldehyde, respectively [21]. 

In 2017, the Dixon group [22] re-evaluated the activity of the 4HBS enzyme (which they previously described) since they had discovered that the gene encoding this enzyme was in fact the same as the *VpVan* gene studied by Gallage et al. [20,23]. Despite extensive efforts, the Dixon group failed to demonstrate any enzymatic activity for VpVAN in various in vitro and in vivo assays, although this could be attributed to a lack of codon optimisation of the *VpVan* gene [22]. Yet, several independent studies using heterologous systems support the ability of VpVAN to convert ferulic acid into vanillin [15,24,25].

Enzymes catalysing the introduction of the C3-hydroxyl group have yet to be fully characterised. Studies on lignin biosynthesis in plants other than *Vanilla* indicate that the 3-hydroxylation could be carried out by a polyphenol oxidase, a dioxygenase, or a cytochrome P450 reductase (CPR). For example, the CPR CYP98A3 from *Arabidopsis*, a 3-monooxygenase, can convert 4-coumaric acid into caffeic acid, albeit very slowly [26]. Recombinant CYP98A3 expressed in yeast metabolised the 5-O-shikimate and 5-O-D-quinate esters of coumarate into the corresponding caffeate conjugates more efficiently than free 4-coumaric acid [27,28]. These findings suggest that 3-hydroxylation may result from multiple reactions requiring several enzymes, including 4-coumaric acid-CoA ligases and hydroxycinnamoyl transferases [27]. Recently, Barros et al. [29] identified and characterised an enzyme from maize that directly catalyses the 3-hydroxylation of 4-coumaric acid to caffeic acid. This enzyme, possessing 4-coumaric acid 3-hydroxylase (C3H) activity, was annotated as ascorbate oxidase (APX1) with, putative orthologs identified in many plants, including in *V. planifolia* (e.g., KAG0466359.1 with 81% identity). The analysis of APX1 deletion mutants in *Brachypodium* and *Arabidopsis* supports its involvement in the early steps of lignin biosynthesis [29]. The ability of CYP98A3 or APX1 to convert 4-hydroxybenzaldehyde to protocatechualdehyde (also known as 3,4-dihydroxybenzaldehyde) was not assessed.

The methylation reaction is catalysed by an O-methyltransferase (OMT) which transfers the methyl group of S-adenosyl-L-methionine (SAM) to a hydroxyl group. This kind of reaction is widespread in plants; for instance, 15 OMTs were identified by sequence analysis in *V. planifolia* [20]. Pak et al. [30] isolated and characterised a multifunctional OMT from *V. planifolia*, showing a preference for caffeoyl aldehyde and 5-OH-coniferaldehyde as substrates over protocatechualdehyde or caffeic acid [30]. The biochemical characterisation of four other O-methyltransferases from *V. planifolia* revealed no link to vanillin synthesis [31,32].

In vanilla pods, vanillin is stored as a nontoxic glucoside, which is more soluble and less toxic than vanillin. UDP-glycosyltransferases (UGTs), which transfer uridine-diphosphate-activated sugars (UDP-sugar) to low-molecular-weight substrates, are responsible for this reaction [10,33]. The timing of this reaction is unknown and glycosylation may occur before the hydroxylation and/or methylation steps. During the curing of vanilla beans, vanillin is released by β-glucosidases, which hydrolyse the glycosidic bond between the sugar and the aglycone [10,34].

How vanillin is biosynthesised in *Vanilla* appears to be still disputed and unclear, possibly because vanillin biosynthesis may follow various routes under varying conditions [17]. Further research is needed to identify the missing enzymes and to clarify the biosynthetic pathway(s). 

## 3. Identification of Genes and Pathways in Vanillin-Producing Microorganisms

The world of microorganisms is vast, and their metabolism is highly versatile. Numerous microorganisms are used for the production of natural food components. Strains of interest are selected based on their growth properties, production potential, and tolerance to high concentrations of both substrates and products. Vanillin-producing microorganisms have been identified for their natural ability to produce vanillin as a catabolic intermediate of phenolic compound degradation pathways. For instance, some species of actinomycetes belonging to the genera *Amycolatopsis*, *Rhodococcus*, and *Streptomyces* were reported to convert ferulic acid into vanillin [35,36,37]. Some species of Gram-negative and Gram-positive bacteria belonging to the *Pseudomonas* and *Bacillus* genera, respectively, were shown to produce vanillin from ferulic acid, eugenol, or isoeugenol [38,39,40,41,42,43]. Finally, some species of fungi, including *Aspergillus niger*, *Phanerochaete chrysosporium,* and *Pycnoporus cinnabarinus*, have been studied for their capacity to produce vanillin by the bioconversion of agricultural and industrial lignin-enriched residues (e.g., waste residues from rice bran [44], fruit peels [45,46], coconut husk [47], and wheat bran [48]). The bioconversion ability to produce vanillin is strain-specific as other species of the same microbial genus are not capable of the same conversions.

**Figure 2:**
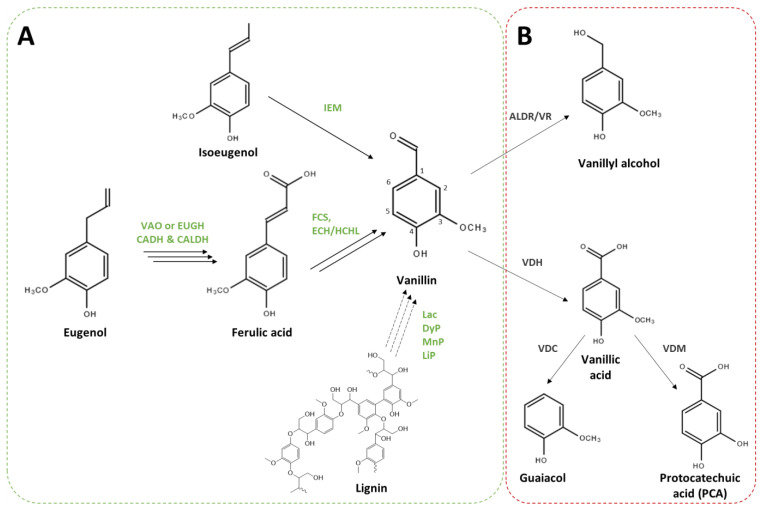
**Vanillin biosynthesis and degradation pathways in vanillin-producing microorganisms.** (**A**) Enzymes involved in vanillin production from different precursors. (**B**) Enzymes involved in vanillin degradation. IEM: isoeugenol monooxygenase; VAO: vanillyl alcohol oxidase; EUGH: eugenol hydroxylase; CADH/CALDH: coniferyl alcohol/aldehyde dehydrogenase; FCS: feruloyl-CoA synthetase; ECH/HCHL: enoyl-CoA hydratase/4-hydroxycinnamate CoA-hydratase/lyase; Lac: Laccase; DyP: dye-decolourising peroxidase; LiP: lignin peroxidase; MnP: manganese-dependent peroxidase; ALDR/VR: aldehyde reductase/vanillin reductase; VDH: vanillin dehydrogenase; VDC: vanillate decarboxylase; VDM: vanillate demethylase.

Genes and enzymes involved in vanillin biosynthesis from phenolic compounds have been characterised in some microorganisms (Figure 2A). Isoeugenol is readily transformed into vanillin through an epoxide-diol pathway by an isoeugenol monooxygenase (gene name: *iem*, enzyme name: IEM) that was found in some *Pseudomonas* strains [49,50,51]. Genes encoding the enzymes involved in the eugenol bioconversion pathway were first identified in *Pseudomonas* sp. HR199 [52]. The transformation of eugenol into ferulic acid comprises three steps: (1) the heterodimeric eugenol hydroxylase (*ehyA*, *ehyB*, EUGH) catalyses the oxidation of eugenol into coniferyl alcohol [52], and this reaction can also be catalysed by a vanillyl alcohol oxidase (*vaoA*, VAO) [53]; (2) and (3) coniferyl alcohol is then oxidised into coniferyl aldehyde and subsequently into ferulic acid by coniferyl alcohol and coniferyl aldehyde dehydrogenases (*calA* and *calB*, CADH and CALDH) [54]. Ferulic acid can then be catabolised by microorganisms, and vanillin is obtained as an intermediate of the catabolic route. Five different ferulic acid catabolic pathways have been proposed in various microorganisms [2,8,11]. The most efficient for vanillin production is the non-β-oxidative deacetylation (CoA-dependent) route (Figure 2A), which requires two enzymes, a feruloyl-CoA synthetase (*fcs*, FCS) and an enoyl-CoA hydratase/4-hydroxycinnamate CoA-hydratase/lyase (*ech*, ECH/HCHL). Genes and enzymes involved in this pathway have been identified and characterised in *Pseudomonas* [55] and *Amycolatopsis* strains [35]. Finally, several microorganisms, including fungi and soil bacteria, have been studied for their ability to degrade lignin and produce vanillin [56]. However, genes and enzymes involved in this bioconversion have yet to be fully characterised [57].

While in *Vanilla,* the biosynthesis of vanillin appears to be connected to the biosynthesis of lignin monomers, in microorganisms, vanillin production seems to be related to phenylpropanoid degradation pathways, in which vanillin is an intermediate. Thus, vanillin might be further catabolised to vanillyl alcohol, vanillic acid, protocatechuic acid (PCA), or guaiacol (Figure 2B). A set of genes implicated in this catabolic pathway was identified in some *Pseudomonas* [38,58,59], *Rhodoccocus* [36], and *Amycolatopsis* [60,61,62] strains.

The various precursors (isoeugenol, eugenol, ferulic acid, and lignin) are not equivalent for the biotechnological production of vanillin. Eugenol and isoeugenol are generated by plants and participate in their defence mechanism. Clove oil is an inexpensive source of both compounds, eugenol being the main compound. However, eugenol is the most challenging substrate due to its high toxicity and low water solubility. Similarly, the major limitation of isoeugenol in vanillin production is its water insolubility. Ferulic acid, a ubiquitous plant constituent, has served as the predominant precursor for the microbial production of vanillin [8]. In plants, ferulic acid primarily forms ester linkages with proteins and saccharides. Free ferulic acid can be obtained from agricultural waste products (such as sugar beet pulp, rice bran, or corn bran) by chemical and enzymatic hydrolysis [8]. Thus, ferulic acid is used as a substrate for natural vanillin production by several companies, including Solvay, Symrise, and Firmenich [8]. Lignin is an essential renewable and inexpensive source of aromatic molecules, and its valorisation can enhance economic and environmental sustainability [63].

## 4. Genetic Engineering Approaches

### 4.1. Overview

**Figure 3:**
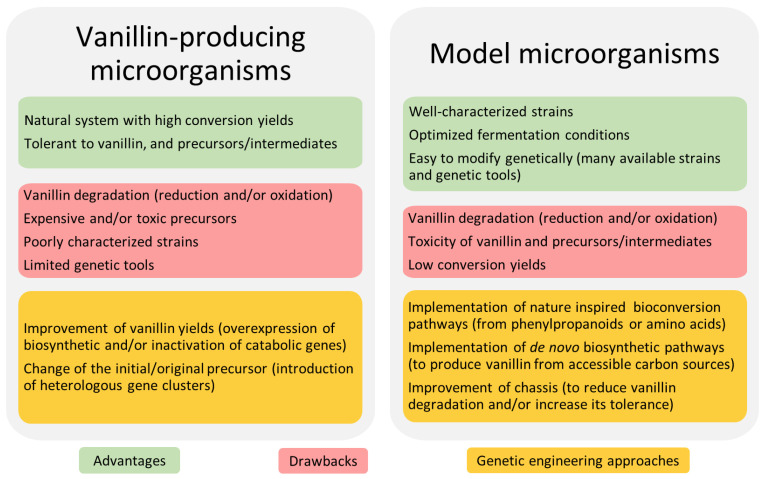
**Vanillin production using different chassis: Advantages; Drawbacks; and Genetic engineering approaches**.

Bio-vanillin can be produced by vanillin-producing or model microorganisms. The advantages and drawbacks of each host chassis for vanillin biosynthesis are summarised in Figure 3. On the one hand, vanillin-producing microorganisms can efficiently produce vanillin and are highly tolerant to vanillin, to the precursors commonly used, and the biosynthetic intermediates. These strains contain the bioconversion pathways for vanillin synthesis from eugenol, isoeugenol, and/or ferulic acid. Vanillin-producing strains used for the industrial bioconversion of ferulic acid into vanillin are not laboratory strains. Most of the time, efficient genetic tools are lacking or limited, hampering their genetic manipulation [60,64]. Moreover, little is known about the native metabolic pathways in which the vanillin biosynthetic genes are involved or the regulation of their expression in these microorganisms [65,66]. 

On the other hand, model microorganisms, such as *Escherichia coli*, *Saccharomyces cerevisiae*, *Corynebacterium glutamicum,* and *Bacillus subtilis*, commonly used in industrial bioconversion processes, benefit from optimised fermentation conditions. A wealth of genetic and synthetic biology tools are available for pathway identification, prediction, reconstruction, and/or implementation in these species [67]. However, vanillin toxicity represents a major problem, as model microorganisms are highly sensitive to this compound and its aldehyde biosynthetic intermediates. Moreover, the intensive optimisation of strains/pathways is often required to reach high vanillin yields.

Despite their different levels of resistance to vanillin, both vanillin-producing and model microorganisms possess resistance mechanisms to reduce the strong antimicrobial activity of vanillin. Common defence mechanisms are the transport of vanillin to an extracellular medium or/and the degradation of vanillin into less toxic compounds. Vanillin degradation in microorganisms can be performed by reductases and oxidases, which are able to convert it into vanillyl alcohol and vanillic acid, respectively, reducing vanillin yields. 

To overcome the aforementioned limitations, genetic engineering strategies are implemented in both types of chassis. In vanillin-producing microorganisms, they aim at: (i) improving vanillin yields by overexpressing biosynthetic genes or by inactivating genes involved in vanillin catabolism to avoid its degradation; and (ii) reducing production costs by introducing genes directing the synthesis of biosynthetic precursors from simple or cheaper substrates. In model microorganisms, genetic engineering approaches aim at: (i) implementing nature-inspired vanillin biosynthetic pathways to produce vanillin from aromatic amino acids; (ii) implementing and optimising de novo vanillin biosynthetic pathways; and (iii) engineering the chassis to develop strains with lower vanillin degradation activity and higher vanillin productivity and tolerance. The following sections describe in detail each of these approaches. 

### 4.2. Genetic Engineering in Vanillin-Producing Microorganisms

#### 4.2.1. Overexpression or Inactivation of Endogenous Genes

Bacteria known to naturally convert phenylpropanoids into vanillin are primarily soil-dwelling bacteria that contribute to the decomposition of organic matters. They belong to the actinomycetes (*Amycolatopsis* spp., *Streptomyces* spp., *Rhodococcus* spp., and *Arthrobacter* spp.) and gammaproteobacteria (*Pseudomonas* spp.) classes. Efforts to optimise the conversion of ferulic acid to vanillin and/or limit vanillin catabolism have essentially focused on the bacteria of these genera. 

**Figure 4:**
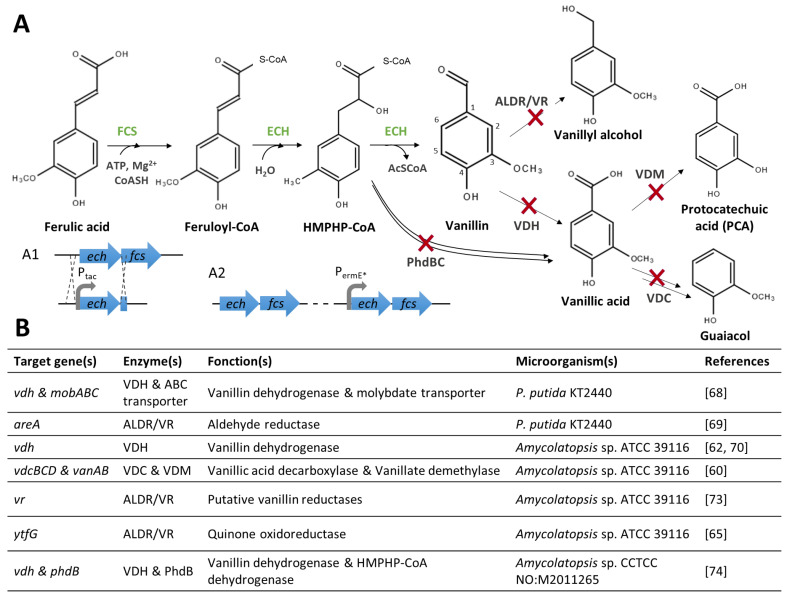
**Optimisation of vanillin biosynthesis from ferulic acid.** (**A**) Ferulic acid catabolic pathway and schematic representation of the constructions used to improve vanillin production; HMPHP-CoA: 4-hydroxy-3-methoxyphenyl-β-hydroxyprpionyl CoA. (**B**) List of genes which have been inactivated in different microorganisms to increase vanillin production [60,62,65,68,69,70,73,74].

In *P. putida* KT2440, both strategies (the optimisation of ferulic acid conversion and the limitation of vanillin degradation) have been carried out (Figure 4). To prevent vanillic acid formation, Graf et al. [68] inactivated *vdh* (encoding a vanillin dehydrogenase) and *mobABC* (coding for a molybdate transporter*).* The inactivation of this transporter reduces the availability of molybdate, a cofactor required by oxidorectudases [68]. After 5 h conversion, the double mutant GN276 converted 6 mM ferulic acid into 4.8 mM vanillin and 0.3 mM of vanillic acid, while the wild-type strain only produced vanillic acid (3.9 mM). Vanillin bioconversion was further optimised by overexpressing *fcs* and *ech* under the control of the strong *tac* promoter (A1 in Figure 4). The resulting strain, *P. putida* GN299, converted nearly 10 mM of ferulic acid into 8.3 mM of vanillin, 0.2 mM of vanillic acid, and 1.1 mM of vanillyl alcohol after 5 h of conversion. To further reduce the vanillic acid formation, the authors deleted three other dehydrogenases, yielding the GN442 strain [68]. Recently, the expression of vanillin reductase genes (identified in the genome of *Pseudomonas* sp. 9.1 and *P. putida* KT2440 by sequence analysis and annotated as aromatic aldehyde reductase, *areA*) in *E. coli* evidenced their capacity to reduce vanillin. Moreover, the deletion of the *areA* gene in the *P. putida* GN442 strain resulted in a decrease in vanillyl alcohol and an increase in vanillin production [69].

In *Amycolatopsis* strains, the optimisation of vanillin production has also been achieved by the overexpression of the genes involved in bioconversion (Figure 4). For instance, the introduction of additional copies of *ech* and *fcs* under the control of the ermE* promoter into the genome of *Amycolatopsis* sp. ATCC 39116 [70] or *Amycolatopsis* sp. ZYL926 [71] resulted in an increase in vanillin production (A2 in Figure 4). Moreover, a recent patent application described an increase in vanillin production from ferulic acid following the inactivation of a gene encoding a ROK-family transcriptional regulator (*ROK-TR1*) in *Amycolatopsis* sp. HM-141 [72]. This regulator might repress, directly or indirectly, the transcription of the genes involved in vanillin biosynthesis, as its overexpression reduces vanillin production compared to the wild-type strain.

Several studies also aimed at limiting vanillin degradation (Figure 4). The genome sequencing of *Amycolatopsis* sp. ATCC 39116 [75] led to the identification of a vanillin dehydrogenase candidate gene *vdh* whose deletion reduced by more than 90% vanillin degradation to vanillic acid [62,70]. Additionally, the deletion of vanillic acid decarboxylase (*vdcBCD*) and vanillate demethylase (*vanAB*) gene clusters showed that in *Amycolatopsis* sp. ATCC 39116, vanillic acid is mainly converted into guaiacol by decarboxylation [60]. However, *vdh* mutants can still produce vanillyl alcohol under fermentation conditions. Researchers from Rhodia Operations used five genes susceptible to encode enzymes reducing vanillin into vanillyl alcohol [73]. These aryl-alcohol dehydrogenases have been named “vanillin reductases” after the validation of their catalytic activity. According to the Rhodia Operations patent, the inactivation of the five *vr* genes yielded an *Amycolatopsis* sp. ATCC 39116 strain that did not produce vanillyl alcohol [73]. In 2018, Meyer et al. [65] reduced the formation of vanillyl alcohol over the entire course of the fermentation by deleting the *ytfG* gene, encoding a putative oxidoreductase. In the same study, authors could also reduce the vanillic acid concentration using a mutant strain deleted for the *vdh* gene and the putative ferulic acid β-oxidation gene cluster (*Δ0689–0695*), including a *phdB* gene encoding a 4-hydroxy-3-methoxyphenyl-β-hydroxypropionyl-CoA (HMPHP-CoA) dehydrogenase [65]. Similar results were recently obtained by Wang et al. [74], who constructed and compared vanillin production in the deletion mutants of *Amycolatopsis* sp. CCTCC NO: M2011265. Compared to the single-mutant *Δvdh*, the double mutant *ΔvdhΔphdB* further increased vanillin and decreased vanillic acid production [74]. 

**Figure 5:**
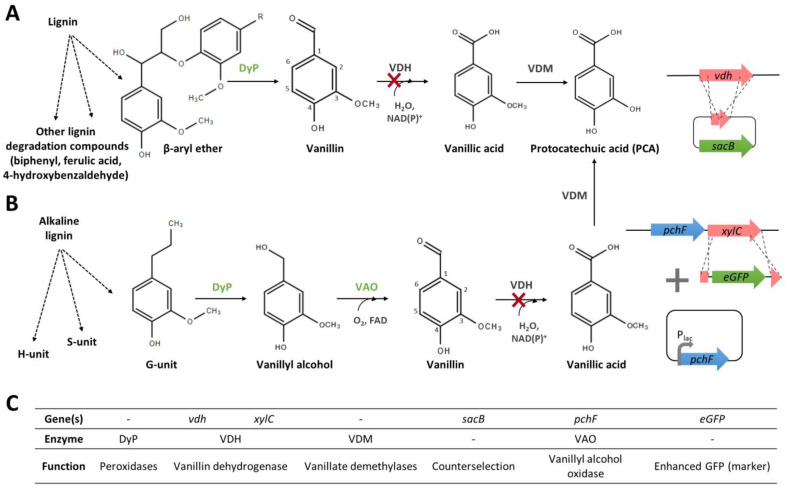
**Microbial vanillin biosynthesis from lignin.** (**A**) Lignin degradation pathway with vanillin as an intermediate in *Rhodococcus jostii* RHA1 and schematic representation of the method used for deletion of *vdh*, in order to increase vanillin production. (**B**) Lignin degradation pathway with vanillin as an intermediate in *Arthrobacter* sp. C2 and schematic representation of the recombinant strain constructed to improve vanillin production. (**C**) Table summarising the function of the genes/enzymes used.

The valorisation of lignin-enriched residues for the production of high-value molecules, including vanillin, is very attractive for environmental, social, and economic reasons [76,77]. Actinomycetes belonging to two genera, *Rhodococcus* and *Arthrobacter*, have been studied for their potential to produce vanillin using lignin derivatives as sole sources of carbon and energy. A sequence analysis of *Rhodoccocus* I24 and PD630 led to the identification of *fcs*, *ech,* and *vdh* genes. The inactivation of *fcs* and *vdh* genes in *Rhodoccocus* PD630 confirmed their involvement in the ferulic acid catabolism and suggested vanillin as an intermediate of this catabolic pathway [36]. The whole genome sequencing of *Rhodococcus jostii* RHA1 [78] allowed the identification and characterisation of genes involved in vanillin catabolism *(vdh* and *vanAB*) by gene disruption and an enzyme activity assay [79]. Therefore, target pathway engineering approaches have been developed to improve vanillin production from lignin. For instance, Sainsbury et al. [80] noticed that a *vdh* deletion mutant strain of *Rhodococcus jostii* RHA1 (Figure 5A) accumulated 96 mg/L of vanillin when grown on a minimal medium containing wheat straw lignocellulose. 

Zhao et al. [81] reported the development of recombinant strains of the lignin-degrading bacterium *Arthrobacter* sp. C2 for the production of vanillin from corn stalks alkaline lignin. According to GC-MS analyses, alkaline lignin is first degraded into three types of monomers (H-, S-, and G-lignin units) through endogenous pathways of the *Arthrobacter* sp. C2 strain (Figure 5B). G-units can be converted to vanillyl alcohol, which is then transformed into vanillin by a vanillyl alcohol oxidase (*pchF*, VAO). Vanillin is further degraded to vanillic acid, which is used as a carbon source after conversion into PCA by a benzaldehyde dehydrogenase (*xylC*, VDH) [81]. The authors have disrupted the *xylC* gene and overexpressed the *pchF* gene under the control of the promoter P_lac_ to optimise vanillin biosynthesis from alkaline lignin (Figure 5B). This recombinant strain produced about 57 mg/L vanillin, while the reference *Arthrobacter* sp. C2 strain produced only trace amounts (<4 mg/L) [81]. Despite the low vanillin yields obtained, these studies showed the potential of microbial approaches for lignin valorisation.

#### 4.2.2. Introduction of Heterologous Genes Directing the Synthesis of Vanillin Precursors

**Figure 6:**
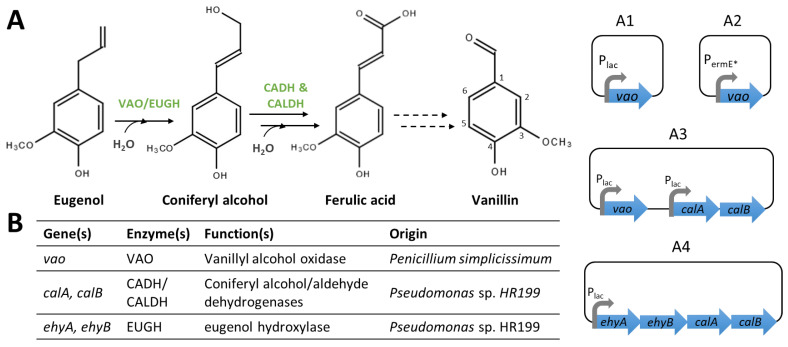
**Genetic engineering approaches allowing the use of eugenol as a precursor of ferulic acid for vanillin bioproduction.** (**A**) Schematic representation of the biosynthetic pathway and the applied constructions. (**B**) Table summarising the function and origin of the genes/enzymes used.

As mentioned before, ferulic acid is quite expensive, and its availability (and price) can vary, so it is desirable to implement pathways that allow the use of economically viable substrates. However, the lack of efficient genetic tools in most vanillin-producing microorganisms makes the genetic manipulations more complex. So far, only the introduction of a ferulic acid biosynthetic pathway from the cheap metabolite eugenol has been described. Thus, the heterologous expression of the *vaoA* gene, from *Penicillium simplicissimum*, under the control of the lac promoter in *Amycolatopsis* sp. HR167 (Figure 6(A1)), using a replicative vector, enables the production of trace amounts of vanillin from eugenol [82]. The expression of the *vaoA* gene under the control of ermE*p in *Streptomyces* sp. (Figure 6(A2)) was also reported for vanillin production from eugenol [83]. These studies showed that the strains used possess endogenous genes encoding enzymes able to convert coniferyl alcohol into ferulic acid and vanillin. However, the *Amycolatopsis* sp. HR167 strain expressing *vaoA* accumulated coniferyl alcohol and coniferyl aldehyde in the medium, suggesting the low specificity of native enzymes for the conversion of coniferyl alcohol into vanillin. To improve vanillin production from eugenol, the authors suggested the additional heterologous expression of the *calA* and *calB* genes in this *Amycolatopsis* sp. HR167 recombinant strain [82].

The introduction of the full biosynthetic pathway from eugenol to ferulic acid has also been tested in other bacteria. For instance, the expression of the *vaoA* gene, from *Penicillium simplicissimum*, and the *calA* and *calB* genes from *Pseudomonas* sp. HR199 in *Rhodococcus opacus* PD630 allowed the production and transient accumulation of ferulic acid from eugenol (Figure 6(A3)) [36]. Similar results were obtained using a recombinant strain of *Ralstonia eutropha* H16 expressing the *ehyA*, *ehyB,* and *calA* and *calB* genes from *Pseudomonas* sp. HR199 (Figure 6(A4)) [84]. Ferulic acid could be purified and then converted into vanillin by vanillin-producing microorganisms.

### 4.3. Genetic Engineering in Model Microorganisms

#### 4.3.1. Implementation of Nature-Inspired Bioconversion Pathways

##### Vanillin from Phenylpropanoids

The second approach to the microbial production of vanillin involves the introduction of the vanillin biosynthetic pathway in model microorganisms. When considering the implementation of phenylpropanoid bioconversion pathways, *Escherichia coli* has served as the primary chassis microorganism. During the past two decades, a number of engineered *E. coli* strains have been developed to produce vanillin from eugenol, isoeugenol, or ferulic acid.

**Figure 7:**
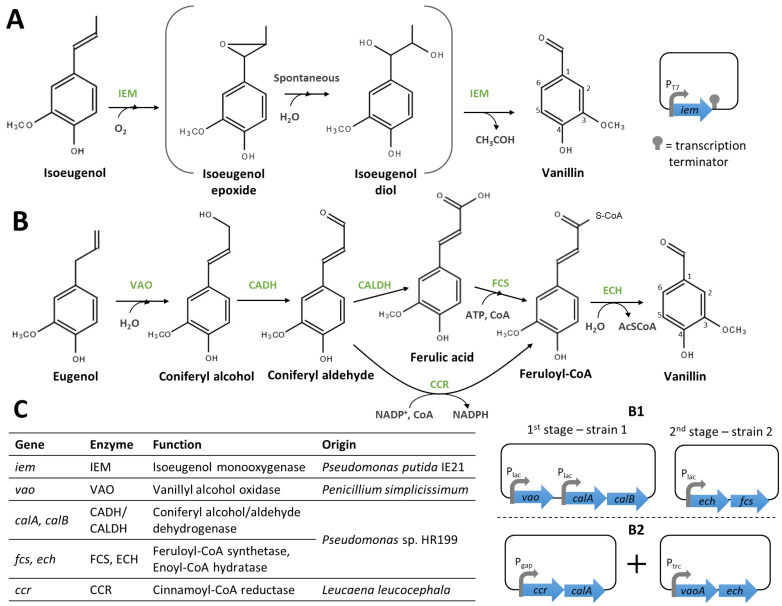
**Vanillin production from eugenol or isoeugenol in *E. coli*.** (**A**) Pathway from isoeugenol and schematic representation of the genetic construction used for its implementation. (**B**) Pathways from eugenol and schematic representation of the genetic constructions used for their implementation. (**C**) Table summarising the function and origin of the genes/enzymes used.

The conversion of isoeugenol via the epoxide-diol pathway requires only an IEM (Figure 7A). Yamada et al. [85] chose to clone the *iem* gene of *Pseudomonas putida* IE27 under the control of the T7 promoter (P_T7_) in *E. coli* BL21(DE3). Resting cells of this recombinant strain produced 186 mM (≈28 g/L) of vanillin from 230 mM of isoeugenol in 6 h [85]. Although the yield obtained in this experiment is very interesting (>80%), the use of resting cells at an industrial level is difficult due to the need for large-volume washing and centrifugation cycles. Recently, Zhao et al. [86] employed semi-rational protein engineering using a protein IEM720 (encoded by a homolog of *iem* isolated from metagenomic DNA) to create a F281Q variant with better enzymatic properties (higher *k*_cat_ and specific activity) than the wild-type enzyme [86]. Thus, protein engineering offers new opportunities for the industrially relevant optimisation of vanillin biosynthetic pathways.

Overhage et al. [87] reported the development of a two-stage process for the biotransformation of eugenol to vanillin (Figure 7B). First, a recombinant *E. coli* XL1-blue co-expressing *vaoA* from *Penicillium simplicissimum* CBS 170.90 and *calA* and *calB* genes from *Pseudomonas* sp. HR199 converted eugenol to ferulic acid with a molar yield >90%. Then, the conversion of ferulic acid to vanillin was tested by co-cultivating this strain with a second one co-expressing *fcs* and *ech* from *Pseudomonas* sp. HR199 (Figure 7(B1)) at a higher temperature to inactivate CALDH (which also possesses vanillin dehydrogenase activity). However, the conversion yield was only 8% and vanillin was further reduced to vanillyl alcohol [87]. Recently, Zhu et al. [88] showed that coniferyl aldehyde inhibits the conversion of ferulic acid into vanillin by FCS and ECH and confirmed that CALDH oxidases vanillin to vanillic acid. To circumvent these drawbacks and simplify the pathway, the authors proposed the use of a cinnamoyl-CoA reductase (CCR) from *Leucaena leucocephala*, which can directly convert coniferyl aldehyde to feruloyl-CoA. The complete pathway, composed by VAO, CADH, CCR, and ECH enzymes, was implemented into *E. coli* BW25113 (Figure 7(B2)), yielding 0.07 mM of vanillin and 1.92 mM of vanillyl alcohol from 2 mM of eugenol [88]. The reduction of vanillin to vanillic alcohol was catalysed by endogenous reductases, whose inactivation improved vanillin yields (see Section Reduction of Vanillin Degradation).

**Figure 8:**
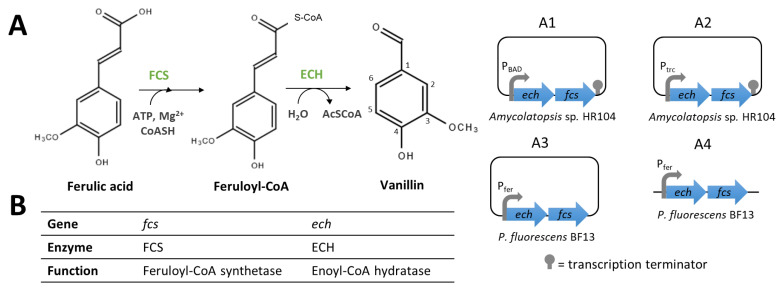
**Vanillin production from ferulic acid in *E. coli*.** (**A**) CoA-dependent non-β-oxidative deacetylation pathway and schematic representation of the construction used for its implementation. (**B**) Table summarising the function of genes/enzymes used. The origin of genes is indicated under the schematic representation of plasmids.

Ferulic acid bioconversion by non-β-oxidative deacetylation (CoA dependent) pathway involves the expression of the *ech* and *fcs* genes (Figure 8). Yoon et al. [89] cloned *ech* and *fcs* genes from *Amycolatopsis* sp. HR104 or *Delftia acidovorans* under the control of the arabinose-inducible promoter (P_BAD_) into the pBAD24 vector. The highest vanillin production (0.58 g/L of vanillin from 1 g/L of ferulic acid) was obtained with the *E. coli* strain carrying the *Amycolatopsis* sp. HR104 genes (Figure 8(A1)) in LB medium [89]. In another study, this group cloned, the *Amycolatopsis* sp. HR104 genes under the control of the *trc* promoter (P*_trc_*) into the pTrc99A vector (Figure 8(A2)). The *E. coli* strain carrying this construct produced 1.1 g/L of vanillin after 48 h of cultivation in 2YT medium [90]. Barghini et al. [91] reported the use of resting cells of *E. coli* JM109 harbouring a low-copy number vector (Figure 8(A3)) based on the RK2 replicon with *ech* and *fcs* genes from *Pseudomonas fluorescens* BF13 under the control of their native promoter (P*_fer_*) to achieve a yield of 0.36 g/L of vanillin [91]. Recently, the same group [92] used an integrative vector to introduce the *ech/fcs* gene cassette into the *E. coli* JM109 chromosome (Figure 8(A4)). The amount of vanillin obtained increased by 1.52-fold compared to the strain harbouring the low copy RK2 derivative vector. An even higher vanillin amount (4.26 g/L, molar yield ≈ 88%) was obtained when the experiment was carried in fed-batch conditions [92]. In the above-mentioned studies, the conversion yield of ferulic acid into vanillin varies between 70 and 88%, with important differences in the final amount of vanillin obtained. The reasons explaining these differences cannot be deduced from these studies, since several parameters (origin of the genes, promoters, expression vectors, strains, culture conditions, etc.) differ from one study to the other. In the future, systematic studies will be needed to establish the contribution of each factor to the final yield. The effective activity of CoA-dependent enzymes requires the constant availability of this cofactor. Therefore, to improve the vanillin yield, some strains carrying vanillin biosynthetic pathways have been engineered to overproduce coenzyme-A (see Section Optimisation of metabolic fluxes to improve cofactor and precursor supplies).

Recently, based on sequence and structure analysis of ECH from *Streptomyces* sp. V1, Ye et al. [93] performed rational protein engineering and generated nine mutants, of which three (F74W, R147Q, and ∆N1-11) exhibited a better vanillin yield than the wild-type enzyme. An in silico analysis could partially explain the negative results obtained by the six other mutants, but suggested that a balance between catalytic activity and enzyme stability must be considered to optimise the vanillin yield [93].

##### Vanillin from Aromatic Amino Acids

**Figure 9:**
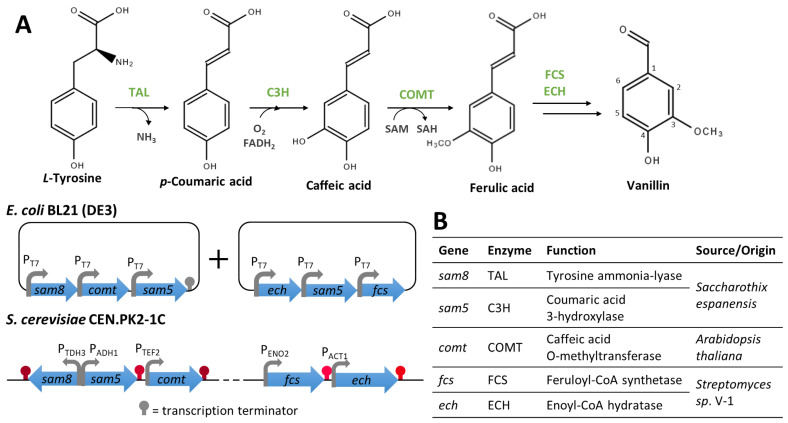
**Vanillin production from L-tyrosine by a ferulate biosynthetic pathway.** (**A**) Schematic representation of the biosynthetic pathway and of the constructions used to implement it in *E. coli* or *S. cerevisiae*. Different transcriptional terminators are represented by different colors. (**B**) Table summarising the function and origin of the genes/enzymes used.

Inspired by the proposed vanillin biosynthetic pathways occurring in *Vanilla* and in vanillin-producing microorganisms, some researchers reported the use of synthetic biology approaches to produce, in model microorganisms, vanillin from aromatic amino acids, which have a lower price and higher availability than ferulic acid. For instance, Ni et al. [94] produced vanillin from accessible carbon sources in *E. coli* by mimicking the ferulate pathway (Figure 9). They assembled a vanillin biosynthetic pathway, previously proposed in an iGEM project [95], comprising five enzymes: TAL, C3H, OMT, FCS, and ECH. In this work, the authors tested two candidate genes encoding enzymes catalysing the conversion of tyrosine to 4-coumaric acid (*RsTAL* and *sam8*) and 4-coumaric acid to caffeic acid (*hpaBC* and *sam5*). Here, only the constructions giving the best vanillin yields are described. The *sam8* and *sam5* genes (from *Saccharothix espanensis*) were cloned in pACYC-Duettogether with a *comt* synthetic gene (codon-optimised for *E. coli*) encoding the caffeic acid O-methyltransferase (COMT) from *Arabidopsis thaliana*. The *ech* and *fcs* genes were amplified from the genomic DNA of *Streptomyces* sp. V-1 and cloned into pET-Duet with an extra copy of *sam5*. The introduction of both vectors in *E. coli* BL21 (DE3) allowed the production of 97 mg/L of vanillin from tyrosine (Figure 9A) [94]. Moreover, the introduction of the same genes in a tyrosine overproducing strain yielded 19 mg/L of vanillin from glucose, 13 mg/L from xylose, and 25 mg/L from glycerol [94].

Qiu et al. [96] recently reported the implementation a similar pathway in *S. cerevisiae* (Figure 9). Only the constructs giving the highest vanillin yields are described here, even if the authors tested three candidate genes encoding enzymes catalysing the conversion of 4-coumaric acid to caffeic acid (CYP199A2, *hpaBC,* and *sam5*). The chosen genes (*sam8* and *sam5* from *Saccharothix espanensis*, *comt* from *Arabidopsis thaliana*, and *fcs* and *ech* from *Streptomyces* sp. V1) were codon-optimised, chemically synthesised, and assembled each with a promoter and a terminator. The selected pathway was introduced simultaneously in two yeast strains, a haploid and a diploid, by the integration of modules into a neutral locus [96]. The amount of vanillin produced by the haploid strain (50.2 µg/L) was higher than the diploid strain, suggesting that the haploid strain is more suitable for vanillin production (Figure 9). Moreover, tyrosine availability in the cell was increased by integrating ARO4 and ARO7 feedback-resistant mutants (encoding a DAHP synthase and a chorismite mutase, respectively) at the delta 22 locus of the haploid strain, leading to the production of 700 µg/L of vanillin [96]. 

**Figure 10:**
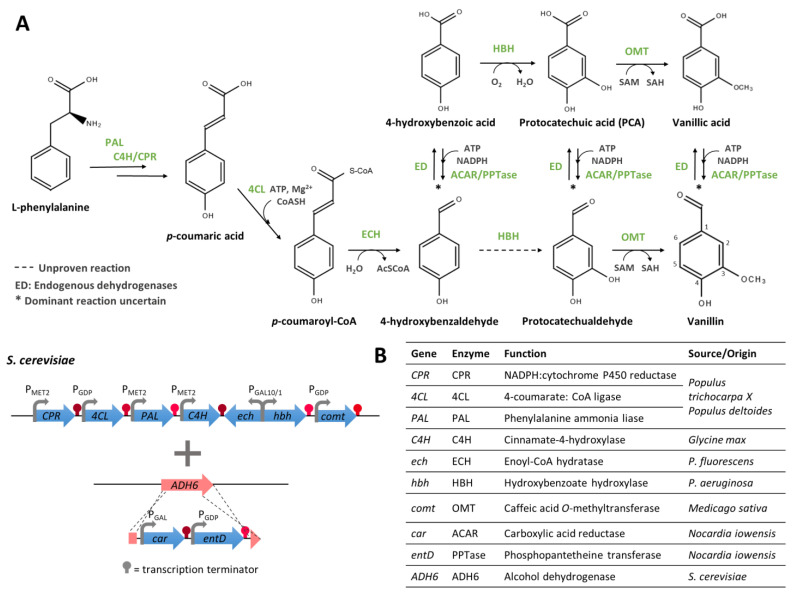
**Vanillin production from L-phenylalanine by a benzoate biosynthetic pathway.** (**A**) Schematic representation of the biosynthetic pathway and of the constructions used to implement it. Different transcriptional terminators are represented by different colors. (**B**) Table summarising the function and origin of the genes/enzymes used.

Another metabolic pathway for the bioconversion of L-phenylalanine to vanillin was proposed by Eviagenics in a patent (Figure 10) [97]. It mimics the benzoate pathway occurring in plants (as shown in Figure 1) [7,10], which had previously been the object of David Michael & Co., Inc. patents concerning the modification of cell plants [23,98]. This pathway comprises six main steps: (i) phenylalanine deamination; (ii) trans-cinnamic acid hydroxylation at position 4; (iii) coumaric acid side-chain reduction; (iv) 4-hydroxybenzoic acid hydroxylation at position 3; (v) PCA O-methylation; and vi) vanillic acid reduction. In its patent, Eviagenics suggests the use of five plant enzymes, namely PAL, C4H, CPR, 4CL, and OMT, to be implemented into a specific yeast cell [97]. The conversion of cinnamic acid into coumaric acid was improved by the co-expression of genes coding for C4H and CPR [97]. To convert coumaroyl-CoA into 4-hydroxybenzaldehyde, a bacterial crotonase is used. Suggested enzymes are, for example, ECH from *P. fluorescens* or from A*zotobacter vinelandii*. To convert the 4-hydroxybenzaldehyde into protocatechualdehyde, the authors described the use of an HBH enzyme instead of the plant 3-monooxygenase. HBH enzymes, coded by *pobA* genes, have been identified in various microorganisms, where they are known to be involved in the catabolism of 4-coumaric acid into PCA [59,99,100,101]. However, like in those natural pathways, it is assumed here that endogenous dehydrogenases would be necessary to first convert the 4-hydroxybenzaldehyde into the corresponding acid. Thus, this pathway is more likely to lead to vanillic acid rather than vanillin (Figure 10). 

Genes encoding the required enzymes were assembled as monocistronic units (i.e., each gene under the control of an inducible promoter and followed by a terminator) in *S. cerevisiae*, yielding the Y00VAN-engineered strain. Such an engineered strain was cultivated under the required inducing conditions and with the addition of phenylalanine as a precursor, which led to 4-coumaric acid, 4-hydroxybenzoic acid, PCA, and vanillic acid being detected in the culture supernatants, but the corresponding aldehydes were not detected [97]. To deal with the undesired oxidation of vanillin and its intermediates (aldehydes) into the corresponding acids, the inventors proposed the replacement of the *ADH6* gene by *car* and *entD* genes from *Nocardia*. These two genes encode an aryl aldehyde carboxylic acid reductase (ACAR, catalysing the reduction of vanillic acid into vanillin) and a phophopantetheinyl transferase (PPTase, responsible for the phosphopantetheinylation of ACAR). The ability of the yeast strain (YOCPVAN) harbouring these heterologous genes to produce vanillin when fed with phenylalanine was not demonstrated [97]. A patent application from Rhodia operations [102] later confirmed the involvement of the host strain endogenous enzyme(s) for the conversion of 4-hydroxybenzaldehyde into 4-hydroxybenzoic acid and described that the engineered strain could produce only 1 µM of vanillin from endogenous phenylalanine.

#### 4.3.2. Implementation of De Novo Bioconversion Pathways

##### De Novo Bioconversion Pathway from Ferulic Acid

Implementing phenylpropanoid bioconversion pathways in *E. coli* allowed us to achieve high yields of vanillin (>70%) from ferulic acid. Moreover, the production of trace amounts of vanillin from aromatic amino acids in *E. coli* and S*. cerevisiae* was achieved by mimicking ferulate and benzoate pathways. However, the industrial implementation of these processes is complicated and energetically costly for the producing microorganism since the applied biosynthetic pathways require ATP and CoA as cofactors, which must be continuously supplied in large amounts to achieve high-yield production. The design of de novo coenzyme A-independent biosynthetic pathways can circumvent this limitation. 

**Figure 11:**
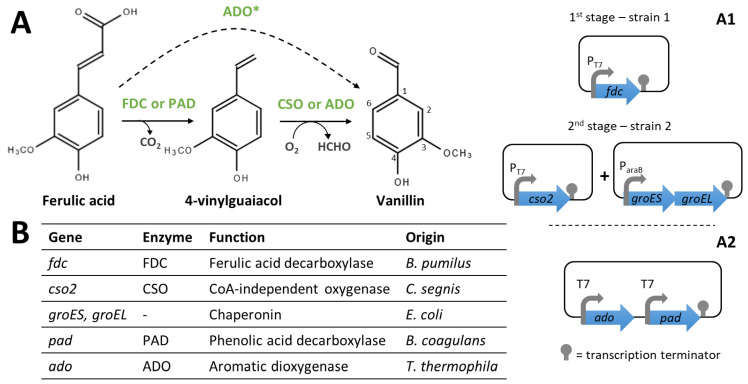
**Vanillin production from ferulic acid by a de novo CoA-independent decarboxylation pathway.** (**A**) Schematic representation of the pathway and of the constructions used. (**B**) Table summarising the function and origin of genes/enzymes used. ADO* represents a modified ADO able to convert ferulic acid directly into vanillin.

In this context, Furuya et al. [103] reported the introduction of a de novo biosynthetic pathway in *E. coli* to produce vanillin from ferulic acid via a CoA-independent route (Figure 11). Here, ferulic acid is first converted to 4-vinylguaiacol by a CoA-independent ferulic acid decarboxylase (FDC), which is then transformed into vanillin by a carotenoid oxygenase (CSO). In this study, 6.8 mM of vanillin was synthetised from 10 mM of ferulic acid using an *E. coli* strain expressing the genes *fdc* and *cso2*. Moreover, researchers demonstrated that the optimal conditions for both reactions are largely different [103]. Later on, the same group obtained a yield of 52 mM of vanillin (from 75 mM of ferulic acid) in a two-stage process using two recombinant *E. coli* BL21 (DE3) (Figure 11(A1)) [104]. The first recombinant strain, harbouring the *fdc* gene from *Bacillus pumilus* under the control of the T7 promoter, completely converted the ferulic acid into 4-vinylguaiacol. The second one, expressing the *cso2* gene (from *Caulobacter segnis*) and overexpressing chaperonins, converted a maximum of 69% of 4-vinylguaiacol into vanillin when the reaction was carried out in a biphasic system to avoid substrate and product toxicity [104]. The co-overexpression of the *groES* and *groEL* genes (encoding the chaperonin GroEL and the cochaperonin GroES) was required to efficiently produce CSO as a soluble protein [103]. 

In both studies by Furuya et al. [103,104], the limiting step was the one catalysed by Cso2, even when optimal conditions (a low temperature and alkaline pH) were used. Thus, the identification of efficient and robust oxygenases is vital to improve vanillin yields using this pathway. Ni et al. [105] identified a thermoresistant aromatic dioxygenase (ADO) from *Thermothelomyces thermophile,* able to convert 4-vinylguaiacol into vanillin. The conversion of ferulic acid to vanillin was tested using *E. coli* BL23 (DE3) co-expressing *ado* and *pad* (PAD, phenolic acid decarboxylase from *Bacillus coagulans*) genes (Figure 11(A2)). This strain could produce 7.1 mM of vanillin from 30 mM of ferulic acid. However, the reaction catalysed by ADO was still the limiting step and endogenous ADHs were very active in these conditions since ~13 mM of 4-vinylguaicol was accumulated and 9.7 mM of vanillic acid was formed. The optimisation of cultures conditions resulted in the production of 27.7 mM of vanillin and limited the accumulation of vanillic acid and 4-vinylguaicol to 0.2 mM and ~2 mM, respectively [105]. The molar vanillin yield obtained in this study (92%) is higher than the ones obtained in studies describing the use of the natural CoA-dependent pathway, so the industrial implementation of CoA-independent pathways may have significant advantages. 

Ni et al. [105] demonstrated that ADO could also catalyse the oxidative cleavage of a C=C bond in other aromatic compounds (e.g., isoeugenol, anethole, and O-methyl isoeugenol). Recently, Fujimaki et al. [106] performed rational protein engineering of ADO, based on in silico analyses, to generate enzyme variants able to catalyse the direct conversion of ferulic acid to vanillin. Variants harboring the F82Y, V332R, and F334R mutations exhibited high catalytic activity on ferulic acid and produced 7.0 mM of vanillin from 10 mM of ferulic acid in 24 h [106].

##### De Novo Bioconversion Pathways from Glucose

**Figure 12:**
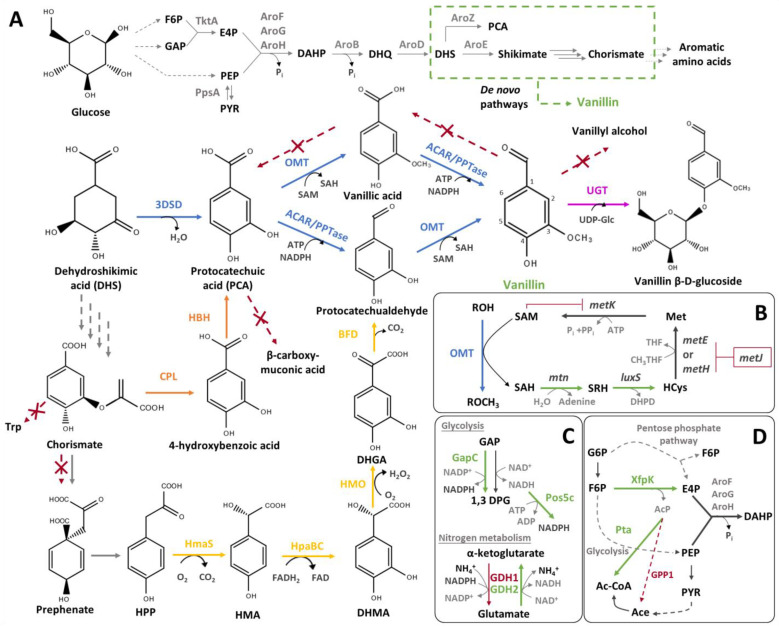
**Vanillin production from glucose by de novo biosynthetic pathways.** (**A**) Vanillin biosynthetic pathways. The arrows representing the reactions are coloured as follows: grey—primary metabolism (e.g., shikimate pathway); blue—conversion of dehydroshikimic acid into vanillin; orange—conversion of chorismate into protocatechuic acid; yellow—conversion of hydroxyphenylpyruvate into protocatechualdehyde; and magenta—conversion of vanillin in vanillin glucoside. Green arrows and enzyme names indicate that the enzymes catalysing the reactions are overexpressed in optimised chassis. Red dotted arrows marked with an X and red enzyme names indicates that the enzyme catalysing the reactions are absent in optimised chassis, following the inactivation of the endogenous genes encoding the corresponding enzymes. (**B**) Strategies to improve the SAM supply for methylation step. (**C**) Modified steps of glycolysis or nitrogen metabolism to improve the NADPH supply. (**D**) Strategies to improve the E4P precursor supply. For panels B, C, and D, the green and red arrows and protein names are used, as in panel A, to indicate overexpression or absence, respectively. Abbreviations of metabolites: Ac-CoA—Acetyl-Coenzyme A; Ace—Acetate; AcP—acetyl phosphate; DAHP—3-deoxy-D-arabino-heptulosonate-7-phosphate; DHGA—3,4-dihydroxyphenylglyoxylate; DHMA—3,4-dihydroxymandelate; DHQ—3-dehydroquinic acid; DHS—3-dehydroshikimic acid; 1,3DPG—3-phospho-D-glyceroyl-phosphate; E4P—erythrose-4-phosphate; F6P—fructose 6-phosphate; GAP—D-glyceraldehyde 3-phosphate; G6P—glucose 6-phosphate; HCys—homocysteine; HMA—hydroxymandelate; HPP—hydroxyphenylpyruvate; Met—Methionine; PEP—phosphoenolpyruvate; Pyr—pyruvate; SAH—S-adenosyl-L-homocysteine; SAM—S-adenosylmethionine; SRH—S-ribosyl-L-homocysteine; Trp—Tryptophan. Abbreviations of proteins: ACAR—Aromatic carboxylic acid reductase; AroB—DHQ synthase; AroD—DHQ dehydratase; AroE—Shikimate dehydrogenase; AroF, AroG, AroH—DAHP synthase feedback-regulated isoenzymes; AroZ, 3DSD—3-dehydroshikimate dehydratase; BFD—Benzoylformate decarboxylase; CPL—Chorismate-pyruvate lyase; GapC—GAP dehydrogenase; GDH1/GDH2—NADP^+^/NAD^+^-dependent glutamate dehydrogenases; GPP1—glycerol-1-phosphate; HBH—Hydroxybenzoate hydroxylase; HmaS—Hydroxymandelate synthase; HMO—Hydroxymandelate oxidase; HpaBC—Two-component flavin-dependent monooxygenase; LuxS—S-ribosylhomocysteine lyase; MetE, MetH—Methionine synthases; MetJ—transcription regulator of methionine biosynthetic gene cluster; MetK—SAM synthetase; Mtn—5′-methylthioadenosine/S-adenosylhomocysteine nucleosidase; OMT—O-methyltransferase; PDC1—pyruvate decarboxylase; Pos5c—cytosol-relocalised NADH kinase; PpsA—phosphoenolpyruvate synthase; PPTase—Phosphopantetheine transferase; Pta—phosphotransacetylase; TktA—Transketolase; UGT—UDP-glycosyltransferase; Xfpk—phosphoketolase.

**Figure 13:**
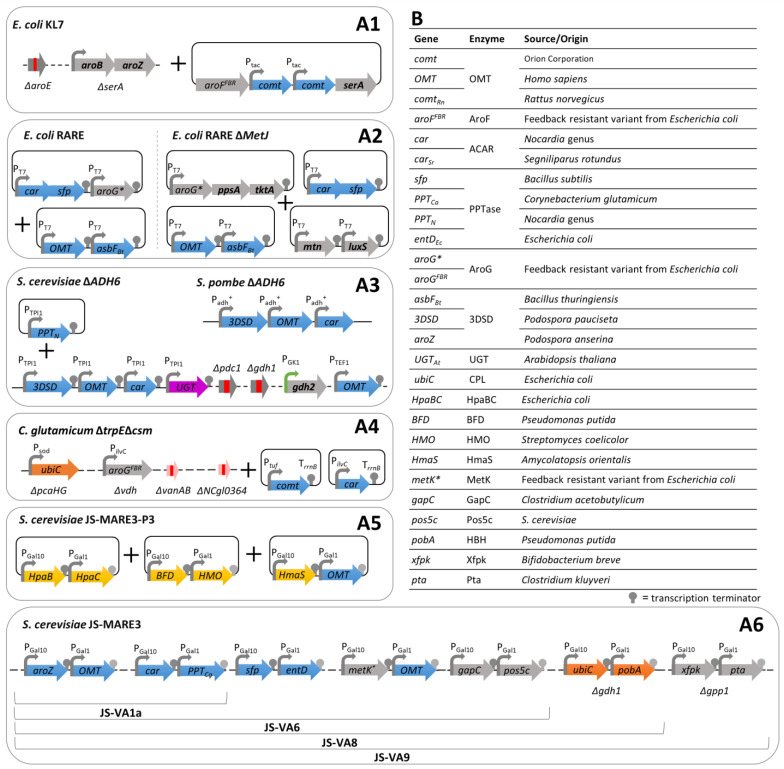
**Implementation of de novo biosynthetic pathways to produce vanillin from glucose in different model chassis.** (**A1**–**A6**) Schematic representation of the genetic constructions used to implement these pathways. Different transcriptional terminators are represented by different colors. Coloured arrows represent genes encoding enzymes involved in different metabolic steps. Blue—conversion of dehydroshikimic acid into vanillin; Orange—conversion of chorismate into protocatechuic acid; Yellow—conversion of HPP into protocatechualdehyde; Magenta—conversion of vanillin in vanillin glucoside; Grey—genes involved in primary metabolism which are overexpressed for chassis optimisation; Red rectangle within an arrow or *∆*—endogenous genes deleted for chassis optimisation; (**B**) Table summarising the origin of the heterologous genes. List of genes deleted in model microorganisms to improve vanillin production: *adh6*—alcohol dehydrogenase; *aroE*—shikimate dehydrogenase; *gdh1*—glutamate dehydrogenase I; *gpp1*—glycerol-1-phosphatese; MARE—multiple-aldehyde reductases; *metJ*—transcription regulator of methionine biosynthetic cluster; *NCgl0324*—aromatic aldehyde reductase; *pcaHG*—protocatechuate dioxygenase; *pdc1*—pyruvate decarboxylase; *serA*—phosphoglycerate dehydrogenase; RARE—multiple-aldehyde reductases; *trpE*—anthranilate synthase; *csm*—chorismate mutase; *vdh*—vanillin dehydrogenase; *vanAB*—vanillate demethylase. List of endogenous genes overexpressed (in bold) for chassis optimisation: *aroB*—DHQ synthase; *aroZ*—3-dehydroshikimate dehydratase; *gdh2*—glutamate dehydrogenase II; *luxS*—S-ribosylhomocysteine lyase; *mtn* –5′-methylthioadenosine/S-adenosylhomocysteine nucleosidase; *ppsA*—phosphoenolpyruvate synthase; *serA*—phosphoglycerate dehydrogenase; *tktA*—transketolase.

The design of de novo coenzyme A-independent biosynthetic pathways allowing the production of vanillin from glucose by the diversion of intermediates from the shikimate pathway (Figure 12) and their introduction in model organisms has been reported in a few studies. For instance, Li and Frost [107] engineered a recombinant *E. coli* strain able to convert glucose into vanillic acid by diverting 3-dehydroshikimic acid (DHS) from the shikimate pathway (Figure 12A Blue). The engineered strain contained a mutated *aroE* gene (encoding a shikimate dehydratase) and an *aroBaroZ* cassette (encoding a DHQ synthase and a 3-dehydroshikimate dehydratase: 3DSD) inserted into the *serA* locus, allowing the accumulation of DHS and promoting its conversion into PCA. This strain also harboured a plasmid carrying an *aroF^FBR^* gene (encoding a feedback-resistant DAHP synthase), two cassettes of the *COMT* gene under the control *tac* promoter, and a *serA* gene (a gene required for L-serine synthesis, which allows one to distinguish recombinant bacteria in minimal medium). The recombinant strain (Figure 13(A1)) produced 3 g/L of vanillic acid and 12.9 g/L of PCA from 20 g/L of glucose. The reduction of these aromatic acids was performed in vitro using a purified ACAR, aryl aldehyde carboxylic acid reductase from *Neurospora crassa*, to synthetise vanillin and protocatechualdehyde, which were then purified by chemical extraction. The same report suggests that the industrial relevance of this metabolic pathway depends on the ability to synthesise vanillin by in vivo reactions implemented in a single microorganism [107]. Later on, Kunjapur et al. [108] generated a *E. coli* RARE strain with a reduced aromatic aldehyde reduction ability (see Section Reduction of Vanillin Degradation), in which the complete metabolic pathway (Figure 12A Blue), including the ACAR step, was introduced, allowing the production of 120 mg/L of vanillin from 12 g/L of glucose (Figure 13(A2)). The vanillin yield was further improved by chassis optimisation (see Section Optimisation of Metabolic Fluxes to Improve Cofactor and Precursor Supplies) [108].

Hansen et al. [109] implemented a similar metabolic pathway in fission and baker’s yeasts. This pathway consists of three enzymes: a 3DSD from *Podospora pauciseta*; an ACAR from the *Nocardia* genus; and an OMT from *Homo sapiens* (Figure 12A Blue). Synthetic genes encoding these enzymes were introduced in alcohol dehydrogenase ADH6 knockout strains to prevent vanillin reduction to vanillyl alcohol. In *Schizosaccharomyces pombe*, the genes were cloned under the control of P*_adh+_* and integrated at the *leu1*^+^ locus. In contrast, in *Saccharomyces cerevisiae*, the genes were cloned under the control of the P*_TPI1_* and directly integrated into the *TPI1* promoter region. In addition, a PPTase gene from *Corynebacterium glutamicum* required for the post-translational activation of ACAR was cloned and expressed under the control of the TPI1 promoter in a low-copy-number-replicating plasmid (Figure 13(A3)). These recombinant strains produce, respectively, 65 and 45 mg/L of vanillin from 20 g/L glucose [109]. *S. pombe* was further improved by introducing a UDP-glycosyltransferase (UGT) from *Arabidopsis thaliana*, which converts vanillin into vanillin β-D-glucoside and consequently circumvents the toxicity of vanillin [109]. Similar pathways to the one described in this work and improved constructs were the subject of several patent applications from industrial companies, such as International Flavors and Fragrances Inc. and/or Evolva [108,109,110].

Instead of diverting 3-dehydroshikimic acid (DHS) from the shikimate pathway, Kim et al. [111] engineered a *Corynebacterium glutamicum* strain to produce vanillin from glucose by the diversion of chorismate (Figure 12A Orange). The strain used was designed to overproduce PCA from chorismate by modulating the primary metabolism through multiple gene deletion, including *trpE* (encoding an anthranilate synthase, involved in tryptophan synthesis), *csm* (encoding a chorismate mutase, responsible for the conversion of chorismite into prephenate), and *pcaHG* (encoding a protocatechuate dioxygenase, responsible for the degradation of PCA into β-carboxy-muconic acid), and the heterologous expression of genes encoding feedback-resistant variants of AroG (*aroG*^FBR^, DAHP synthase) and CPL (*ubiC*, chorismate-pyruvate lyase) from *E. coli*. The conversion of PCA to vanillin involved two enzymes: an ACAR from the *Nocardia* genus and a mutated OMT from *Rattus norvegicus.* Synthetic genes encoding these enzymes were introduced in compatible self-replicative plasmids and transferred into the host strain (Figure 13(A4)). Vanillin could only be produced when the genes *vdh*, *vanAB,* and *NCgl0324* (encoding a putative aromatic aldehyde reductase) were also deleted to prevent vanillin degradation [111].

Recently, Mo and Yuan [112] explored, in a *S. cerevisiae* MARE strain, with a minimal aromatic aldehyde reduction ability (see Section Reduction of Vanillin Degradation), the implementation of these two de novo bioconversion pathways from the shimikate pathway: the one diverting DHS (Figure 12A Blue), previously applied in a *S. cerevisiae* strain [109,110,113,114], and the one based on the chorismate deviation for the overproduction of PCA, previously applied in *Corynebacterium* [111] (Figure 12A Orange); but also of a third one, diverting hydroxyphenylpyruvate (HPP) from the prephenate pathway. This third biosynthetic pathway from glucose to vanillin is based on the hydroxymandelate degradation pathway for the overproduction of protocatechualdehyde (Figure 12A Yellow), and had previously been implemented by the same group in *E. coli* [115,116]. Here, the hydroxymandelate degradation pathway consisted of four enzymes: a hydroxymandelate synthase (HmaS) from *Amycolatopsis orientalis*, a two-component flavin-dependent monooxygenase (HpaBC) from *E. coli*, a hydroxymandelate oxidase (HMO) from *Streptomyces coelicolor,* and a benzoylformate decarboxylase (BFD) from *Pseudomonas putida* [112]. The plasmid-based expression of this pathway confirmed its functionality in *S. cerevisiae* MARE, yielding 20 mg/L of vanillin (Figure 13(A5)). Mo and Yuan [112] also proposed the implementation of dual precursor biosynthetic pathways. First, a vanillin biosynthetic pathway diverting DHS was implemented and optimised, yielding the JS-VA6 strain (Figure 13(A6)), which produced 217 mg/L of vanillin from 20 g/L of glucose (see Section Optimisation of Metabolic Fluxes to Improve Cofactor and Precursor Supplies). Then, one of the two other de novo biosynthetic pathways were introduced into the JS-VA6 strain. It appeared that the hydroxymandelate degradation pathway for the overproduction of protocatechualdehyde was not compatible since the ACAR activity seemed to be repressed by hydroxymandelate analogues [112].

Alternatively, the compatibility of the chorismate deviation for the overproduction of PCA was tested by the integration of *ubiC* from *E. coli* and *pobA* (encoding hydroxybenzoate hydroxylase) from *P. putida* at the *gdh1* (encoding a glutamate dehydrogenase) locus to further reduce NADPH consumption. The resulting strain JS-VA8 increased vanillin production (262 mg/L) compared to the parental strain JS-VA6 (Figure 13(A6)).

##### From PET Monomers

**Figure 14:**
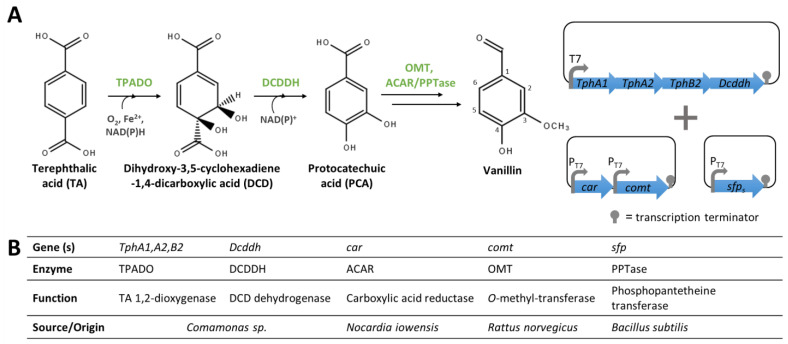
**Implementation of a de novo biosynthetic pathway to produce vanillin from a PET-derived monomer (terephthalate acid).** (**A**) Schematic representation of the biosynthetic pathway and the used constructions to produce vanillin in *E. coli*. (**B**) Table summarising the function and origin of the genes/enzymes used.

A circular economy aims at minimising adverse environmental impacts of manufacturing. In this context, the use of plastic waste to obtain value-added products has attracted significant attention in recent years [117,118]. Polyvinyl alcohol and polyethylene terephthalate (PET) are polymers with widespread applications (e.g., textile industry, pigment binder, and plastic bottle production). However, these polymers are important pollutants and new methods to degrade and valorise them are required for an efficient circular economy [117,118]. Mejia et al. [119] have identified a possible enzyme implicated in the bioconversion of polyvinyl alcohol to vanillin by *Phanerochaete chrysosporium*. Recently, Sadler and Wallace [120] reported the development of an engineered *E. coli* for the direct bioconversion of terephthalic acid (TA), a PET-derived monomer, into vanillin. To this end, they implemented in *E. coli* RARE a de novo pathway comprising four steps (Figure 14). First, the *thpA1*, *tphA2*, *tphB2,* and *dcddh* genes, encoding the enzymes involved in the conversion of TA into PCA, were assembled in a single operon under the control of the T7 promoter in pET21. The *comt* and *car* genes, required for the transformation of PCA to vanillin, were each cloned under the control of the T7 promoter in pRSF-Duet. In addition, a third plasmid comprising the *sfp* gene, encoding a PPTase, was constructed [120]. Cells co-transformed with all three vectors (Figure 14) produced 0.76 mg/L of vanillin from 5 mM of TA. After extensively optimising the bioconversion conditions (such as the culture media, pH, temperature, and the addition of the cofactor’s precursors), a maximum vanillin titre of 119 mg/L was obtained [120].

#### 4.3.3. Engineering of Chassis

##### Reduction of Vanillin Degradation

The major barrier to use *Escherichia coli* and *Saccharomyces cerevisiae* for vanillin biosynthesis is the rapid conversion of vanillin into undesired vanillyl alcohol by several endogenous enzymes. The degradation of desired aldehydes into undesired alcohols can be minimised by the deletion of alcohol dehydrogenases (ADH), aldoketo reductases (AKR), and/or aldehyde reductases (ALDR). However, it may be difficult to completely prevent vanillin reduction due to the catalytic promiscuity and presence of multiple candidate enzymes. Kunjapur et al. [108] generated a *E. coli* RARE strain, with a reduced aromatic aldehyde reduction ability, by the rational target deletion of six genes, encoding three AKR and three ADH, based on the reported activities of these enzymes on benzaldehyde. This *E. coli* RARE strain carrying a de novo vanillin biosynthetic pathway (Figure 12A Blue) and a feedback-resistant form of *E. coli* AroG (aroG*, DAHP synthase) produced about 120 mg/L of vanillin from glucose (Figure 13(A2)), while the wild-type strain only produced trace amounts of vanillin [108]. Likewise, Zhu et al. [88] improved vanillin production from eugenol (Figure 7(B2)) by about 8-fold by the inactivation of two ALDRs (*yjgB* and *yqhD*) and a choline dehydrogenase (*betA*) in *E. coli* BW25113.

In *S. cerevisiae*, Hansen et al. [103] performed a genome-wide search of enzymes with vanillin reductase activity (ADH, AKR, and/or ALDR) and selected 29 candidates to study their ability to reduce vanillin. The analysis of the deletion mutants showed that ADH6 was the most active vanillin reductase in *S. cerevisiae*. Thus, the authors constructed an *adh6* mutant strain for vanillin biosynthesis and suggested that the inactivation of additional reductases would result in a significant increase in vanillin production [103]. Later on, the same and/or additional vanillin reductase candidates were investigated by simple and/or multiple deletions [102,113,114]. Overall, these studies explored 36 candidates and showed that multiple incidences of the inactivation of enzymes with vanillin reductase activity is necessary to efficiently reduce the degradation of vanillin into vanillyl alcohol in *S. cerevisiae* [102,109,113,114]. Mo and Yuan [112] recently designed and constructed *S. cerevisiae* MARE strains, with a minimal aromatic aldehyde reduction ability, in which more than 10 oxidoreductase genes were inactivated. The JS-MARE3 strain was obtained by the deletion of 12 genes, encoding five ADH, four AKR, and three ALDR. To confirm the potential of this yeast platform for the de novo biosynthesis of vanillin, the de novo biosynthetic pathway via DHS was tested (Figure 12A Blue). Here, this pathway was constituted by 3DSD from *Podospora anserina* (*aroZ* gene), OMT from *Homo sapiens*, ACAR from *Segniliparus rotundus,* and PPTase from *Nocardia*. JS-VA1a (Figure 13(A6)) was obtained by the integration of genes into the JS-MARE3 chromosome, and produced 104 mg/L of vanillin with no detection of vanillyl alcohol, contrarily to the control strain, in which vanillyl alcohol was accumulated and only a trace amount of vanillin was found [112].

##### Optimisation of Metabolic Fluxes to Improve Cofactor and Precursor Supplies

CoA-dependent vanillin biosynthetic pathways require the constant availability of this cofactor to achieve high-yield vanillin production. Lee et al. [121] designed a CoA regeneration system in *E. coli* BW25113 by the overexpression of *gltA* and the deletion of *icdA* genes. The implementation of this CoA regeneration system enhanced vanillin production from ferulic acid by 2.8 times, with the molar conversion yield increasing from 34% to 94% compared to the parental strain (Figure 8(A2)). The overexpression of *gltA*, encoding a citrate synthase, increases the conversion of acetyl-CoA (generated during vanillin biosynthesis from ferulic acid) and oxaloacetate into CoA and citrate, respectively. The deletion of *icdA*, encoding an isocitrate dehydrogenase, results in isocitrate flowing through the glyoxylate bypass, a shorter route than the tricarboxylic acid cycle to obtain oxaloacetate [121]. Similarly, Zhu et al. [88] improved the CCR activity and, consequently, vanillin production from eugenol (Figure 7(B2)) by the overexpression of *gltA* and the deletion of *ptsH*, encoding a phosphocarrier protein whose inactivation leads to phosphoenolpyruvate accumulation, stimulating the conversion of acetyl-CoA into CoA and ATP.

To achieve a better vanillin yield, the identification of the limiting steps in de novo vanillin biosynthetic pathways and their optimisation by diverse strategies are essential. In 1998, Li and Frost [107] reported that the improvement of the methylation step was essential for the do novo biosynthesis of vanillin and suggested that S-adenosyl-L-methionine (SAM, a cofactor required for methylation) availability may be limiting in vivo. Later, aiming to improve vanillin yields, Kunjapur et al. [122] increased the availability of DHS precursors and SAM. For that, first, the authors overexpressed the *ppsA* and *tktA* genes (encoding a PEP synthase and transketolase, respectively) to increase the formation of DAHP in *E. coli* RARE. Then, they designed and implemented a SAM regeneration system (Figure 12B) based on the deletion of *metJ* (a transcription regulator of the methionine biosynthetic cluster) and the overexpression of the native *mtn* and *luxS* genes, encoding a 5′-methylthioadenosine/S-adenosylhomocysteine nucleosidase and a S-ribosylhomocysteine lyase, respectively. The resulting strain (Figure 13(A2)) synthetised 256 mg/L of vanillin, and its supplementation with methionine (SAM precursor) resulted in 419 mg/L of vanillin [122].

To avoid vanillin toxicity and improve vanillin yields in *S. cerevisiae*, Brochado et al. [123] expressed the *UGT* gene and applied an in silico metabolic modelling analysis to identify bottlenecks in the metabolic flow for vanillin β-D-glucoside biosynthesis. Based on this analysis, two genes, *PDC1* (pyruvate decarboxylase) and *GDH1* (glutamate dehydrogenase), were identified as target genes whose inactivation might improve vanillin β-D-glucoside production. The deletion of these genes was predicted to increase the availability of ATP and NADPH [124], two cofactors required for vanillin biosynthesis. In addition, researchers overexpressed GDH2 to ensure sufficient nitrogen uptake (Figure 12C). In batch cultivation, a 1.6-fold higher synthesis of vanillin β-D-glucoside was obtained using this engineered strain (Figure 13(A3)) in comparison to the parental strain (expressing the *UGT* gene) [123]. Despite this yield improvement, the accumulation of PCA and protocatechualdehyde intermediates suggested a limiting step of downstream PCA formation [123]. Brochado and Patil [125] reduced the accumulation of protocatechualdehyde and increased the production of vanillin β-D-glucoside by up to 380 mg/L by overexpressing the OMT gene in the previously engineered strain (Figure 13(A3)). [125]. 

Later on, the implementation of the de novo biosynthetic pathway via DHS into the *S. cerevisiae* JS-MARE3 chromosome by Mo and Yuan [112] resulted in 104 mg/L of vanillin and a high accumulation of PCA (375 mg/L), indicating that ACAR and/or OMT reactions are limiting steps. To improve the production of vanillin, the authors further conducted several successive genetic modifications aiming to: (1) improve the phosphopantetheinylation of ACAR by the additional expression of two PPTase from *Bacillus subtilis* and *E. coli* (*Sfp* and *EntD*, respectively); (2) increase the amount of NADPH and ATP cofactors by the introduction of NADP-dependent glyceraldehyde-3-phosphate dehydrogenase (GapC) from *Clostridium acetobutylicum* and a cytosol-relocalised NADH kinase (Pos5c) from *S. cerevisiae* (Figure 12C); and (3) improve the SAM supply and methylation step by the integration of a mutant metK* from *E. coli*, encoding a SAM synthetase variant (I303V, with higher activity and reduced product inhibition) together with an additional copy of OMT (Figure 12D). The resulting strain JS-VA6 (Figure 13(A6)) produced 217 mg/L of vanillin and significantly reduced PCA accumulation (≈200 mg/L) compared to the parental strain JS-VA1a [112]. Additionally, a dual precursor biosynthetic pathway was implemented and the supply of D-erythrose 4-phosphate, a precursor of DHS, was improved by the introduction of two heterologous genes, *xfpk* from *Bifidobacterium breve* and *pta* from *Clostridium kluyveri* (encoding a phosphoketolase and a phosphotransacetylase, respectively), at the *gpp1* (encoding a glycerol-1-phosphate) locus to minimise acetate formation (Figure 12D). The final strain JS-V9 (Figure 13(A6)) produced 352 mg/L of vanillin, which represents the highest vanillin yield from glucose in yeast [112].

##### Improvement of Vanillin Tolerance

A major problem when using model microorganisms as cell factories for vanillin production is the toxicity of this compound. Vanillin, bearing an aldehyde functional group, is highly reactive, causing an accumulation of reactive oxygen species (ROS). ROS mediates the oxidative degradation of unsaturated fatty acids on the membrane, which leads to cell death due to the disruption of the membrane integrity and the production of highly toxic aliphatic aldehydes derived from endogenous lipids [126]. Moreover, in *S. cerevisiae*, it was reported that vanillin represses the translation due to incorrect ribosome assembly, which causes the accumulation of processing bodies and stress granules composed of non-translating mRNAs and various proteins [127]. As described before, common vanillin resistance mechanisms for microorganisms are vanillin efflux and/or its degradation into less toxic compounds such as vanillyl alcohol and vanillic acid. Vanillin-tolerant yeast strains were obtained by over-expressing genes encoding reductases or dehydrogenases involved in vanillin detoxification [128]. However, as it decreases vanillin yields, such approaches are not suitable for vanillin production. 

Recently, transcriptomic, proteomic, and metabolomic studies were employed to understand the vanillin metabolic network and elucidate vanillin resistance mechanisms in microbes [57,58,65,66,129]. For instance, in a proteomic study supported by multiple physiological experiments and mutant analyses, Pattrick et al. [130] have identified that an RND efflux gene (*acrD*) and two aromatic acid efflux genes (*aaeA* and *aaeB*) are involved in vanillin resistance in *E. coli*. Similarly, vanillin tolerance was increased in *S. cerevisiae* by the deletion of the YRR1 gene, which upregulated genes encoding ABC transporters, dehydrogenases, and proteins involved in ribosome biogenesis [131]. Therefore, increasing vanillin efflux by overexpressing vanillin-dedicated membrane transport proteins might be an effective strategy for engineering vanillin-tolerant hosts for vanillin synthesis. However, the identification of vanillin-specific transporters is difficult due to the presence of multiple transport proteins and their wide substrate specificity. For instance, dozens of transporter genes have been identified in vanillin-producing organisms (e.g., *P. putida* KT440, *Amycolatopsis* sp. ATCC 39116) [65,132]. Thus, rapid strategies to increase vanillin resistance include vanillin recovery using adsorbent resins [133] or a solid–liquid two-phase partitioning bioreactor system [134]. In a recent study, Li et al. [135] screened aromatic compound transporter candidate genes from *P. putida* to identify those whose were efficient substrate importers and product exporters in a *E. coli* recombinant strain producing vanillin from ferulic acid. Then, based on the screening results, they constructed an autoregulatory bidirectional transport system by co-expressing the genes *todX* (encoding a ferulic acid importer) and *pp_0179* (encoding a vanillin transporter) under the control of the vanillin-inducible promoter *ADH7* from *Saccharomyces paradoxus* [135]. This system significantly enhances the substrate uptake efficiency while alleviating the vanillin toxicity issue, thus allowing higher vanillin production.

## 5. Discussion

**Figure 15:**
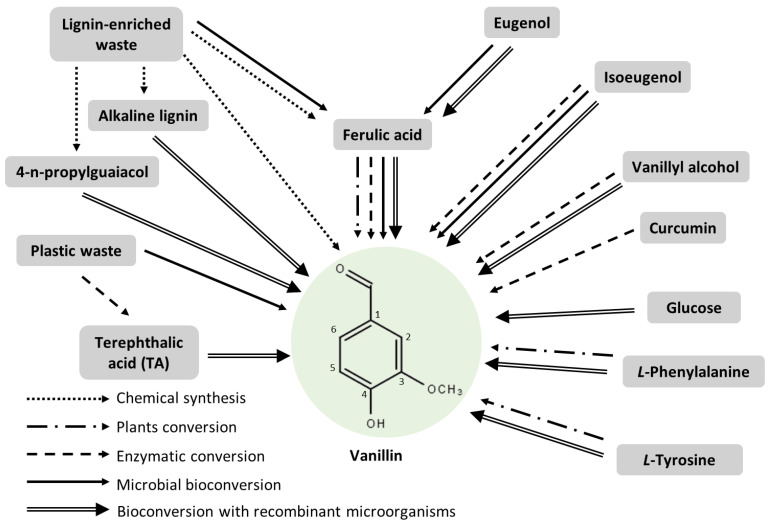
Different routes for vanillin production.

Vanillin is the flavouring agent most used in the world. Besides extraction from vanilla pods, vanillin can be produced using chemical or biotechnological approaches (Figure 15). According to flavourists, vanillin alone does not constitute vanilla flavour, characterised by floral, smoky, sweet, butter, tobacco-like, and woody notes [136]. Therefore, bio-vanillin will not replace vanilla extract, but might replace synthetic vanillin with a “natural flavour” at an affordable price. Several biotechnological methods, summarised in Figure 15, have been established and optimised to produce bio-vanillin. Biotechnological methods have the advantage of using low quantities of safe chemicals and, therefore, are environmentally friendly. These methods have been developed based on the knowledge acquired on the reactions/enzymes involved in vanillin biosynthesis in *Vanilla* and vanillin-producing microorganisms. They can be classified as plant-, enzyme-, or microorganism-based approaches according to the platform used for production [11]. Plant-based approaches involve the use of plant tissues to biosynthesise vanillin. Some examples reported the heterologous gene expression in beetroot [137], chilli pepper [24], and rice [25] of genes involved in the conversion of ferulic acid to vanillin. However, the yield of vanillin production was insignificant due to the slow growth of plant cells and the weak expression of the biosynthetic enzymes. Enzyme-based approaches generate better vanillin yields and have been described for vanillin production from ferulic acid [104,138], curcumin [139], vanillyl alcohol [140,141], and isoeugenol [142]. However, the high costs associated with enzyme production, purification, and formulation reduce the use of these approaches to produce vanillin industrially because of their low economic viability [11]. In this context, to bypass enzyme purification costs, some studies proposed the use of whole-cell catalysis for vanillin production. For instance, the conversion of 4-n-propylguaiacol (4PG), one of the main components of lignin oil, into vanillin was reported using recombinant *E. coli* resting cells [143]. First, 4PG was transformed into isoeugenol, using an *E. coli* strain expressing a bacterially engineered eugenol oxygenase. Then, a second strain carrying a non-heme-dependent dioxygenase mutant was used to convert isoeugenol into vanillin [143]. In another study, lignin-based vanillyl alcohol was converted into vanillin using recombinant *E. coli* resting cells expressing 5-hydroxymethylfurfural oxidase [144]. 

Microorganism-based approaches for bio-vanillin production are more attractive due to the metabolic versatility, the ease of the genetic manipulation of microorganisms, and the low production cost. Different genetic engineering approaches have been explored to improve endogenous vanillin biosynthetic pathways or implement exogenous ones in microorganisms. First, vanillin-producing bacteria have been genetically modified to improve the bioconversion of ferulic acid into vanillin. On the one hand, the overexpression of endogenous genes involved in vanillin biosynthesis (*fcs* and *ech*) allowed an improvement of the final vanillin concentration and of the molar yield (Table 1) [71]. An even better molar yield (95%) was obtained after the deletion of *vdh* and the overexpression of *ech* and *fcs* genes in *Amycolatopsis* sp. ATCC39116 [70]. These approaches were efficient since the molar yields were very satisfactory (close to 100%). On the other hand, an improvement in vanillin production could be achieved by deleting a transcriptional regulator belonging to the ROK-family in a bacterium belonging to the *Amycolatopsis* genus named *Amycolatopsis* sp.

Even if not much is known about native regulation in these bacteria, approaches modifying the gene expression regulation might significantly affect vanillin production.

Secondly, the heterologous expression of genes involved in vanillin biosynthetic pathways from phenylpropanoids has been achieved in *E. coli* [85,86,87,89] and lactic acid bacteria [145]. Eugenol was converted to ferulic acid by genetically engineered *E. coli* with a molar yield >90%; however, further transformation to vanillin was inefficient (Table 1) [87]. Several attempts to improve ferulic acid bioconversion in *E. coli* have been tried and a final vanillin concentration of 4.3 g/L was obtained by the integration of the required genes (*fcs* and *ech*) into the chromosome [92]. An even better yield (5.14 g/L) was obtained in *E. coli* after the implementation of a CoA regeneration system, allowing a constant supply of this cofactor [121]. These investigations also allowed us to study the enzymes involved in ferulic acid bioconversion and to identify bottlenecks, including the substrate and product toxicity, the promiscuity of enzymes, and the genetic instability of recombinant strains, and remedy it. Thus, resting cells have been used to bypass isoeugenol toxicity and produce a high concentration of vanillin (28.3 g/L) [85]. The use of scaffolds to co-localise enzymes and avoid the loss of intermediates has been proposed [146].

Ferulic acid can be efficiently converted into vanillin by non-recombinant vanillin-producing strains and by recombinant strains. However, the availability of ferulic acid is low and variable, and the price of it can fluctuate significantly, increasing production costs. Thus, the biosynthesis of vanillin from simple carbon sources (such as sugars or amino acids) or alternative sources (e.g., agro-waste and plastic waste) has been investigated to avoid this problem and reduce costs. The implementation of the ferulate [94,96] and benzoate [97] biosynthetic pathways for vanillin production using aromatic amino acids has been reported in *E. coli* and yeast. Moreover, de novo biosynthetic pathways have been designed to produce vanillin from glucose in specific *E. coli* [108], *Corynebacterium glutamicum* [111], and yeast strains [109,112]. In addition, a de novo pathway was established in *E. coli* to produce vanillin from terephthalic acid (TA), a PET-derived monomer [120]. In all cases, even after extensive optimisation, yields were below 0.5 g/L (Table 1), suggesting that optimisations are still required to achieve industrially attractive yields. 

Explanations for these low yields include poor gene expression, low enzyme activity and specificity, low cofactor availability, the deviation of intermediates to undesired pathways, and vanillin toxicity. The enzymes involved in these pathways have low activity and selectivity. For instance, the enzymes FCS and ECH, which convert ferulic acid to vanillin, can also convert 4-coumaric acid and caffeic acid into 4-hydroxybenzaldehyde and protocatechualdehyde, respectively [59,147]. Moreover, enzymes catalysing a 3-hydroxylation reaction, such as cytochromes P450 or monooxygenases, have been reported to have a broad spectrum of substrates, acting on different phenolic compounds [148,149]. Furuyra et al. [150] performed site-directed mutagenesis in the gene encoding CYP199A2 from *Rhodopseudomonas palustris* to obtain cytochrome P450 mutants with improved catalytic properties. A F185L mutant exhibits 5.5-fold higher activity on 4-coumaric acid than the wild-type enzyme. *E. coli* cells expressing this mutated enzyme produced 2.5 g/L of 4-coumaric acid in 24h [150]. Therefore, identifying and characterising new enzymes [151,152], or mutagenesis of the existing ones [153], to increase their activity and selectivity is a prerequisite to improving vanillin yields. Vanillin-biosensors, enabling the efficient and rapid detection of vanillin facilitates the screening of strains for vanillin production optimisation, allowing the development of high throughput methods. For example, vanillin-inducible promoters were used to construct a vanillin-sensing *E. coli*, which was used to screen new enzymes able to degrade lignin and convert its derivates to vanillin [88,154]. Similarly, vanillin-sensing *E. coli* was designed based on the VanR-VanO system and has been used to select an efficient OMT for the conversion of protocatechuate to vanillate [155]. Recently, the vanillin-inducible promoter *ADH7* from *S. paradoxus* was used to screen aromatic transporter candidates and to construct a vanillin-induced bidirectional transport system in *E. coli* [135].

Compared to vanillin-producing microorganisms, *E. coli* and yeast are highly sensitive to vanillin. Common vanillin detoxification mechanisms are based on vanillin degradation, which is not desired during vanillin production processes. Thus, suitable recovery strategies have mainly prevented vanillin toxicity [133,134]. The overexpression of vanillin-dedicated membrane transport proteins might be an effective genetic engineering strategy to develop vanillin-tolerant strains [130,156]. In this case, vanillin would not be degraded, but transported outside the cell, preventing its toxic effect and facilitating its recovery. Another possible strategy to bypass vanillin toxicity could be the implementation of de novo biosynthetic pathways in vanillin-producing microorganisms, which are highly tolerant to vanillin and its biosynthetic intermediates. However, the genetic engineering tools to efficiently manipulate these microorganisms [60,64,70] are less developed than in *E. coli* and yeast, and little is known about the metabolism of vanillin-producing microorganisms. Therefore, developing additional tools is essential to implement de novo biosynthetic pathways in these microorganisms effectively. Moreover, further pathway improvements might require genomics, transcriptomics, proteomics, and metabolomics studies.

Ferulic acid is a ubiquitous plant constituent. Yet despite its high abundance in nature, its recovery from plant cell walls (or agricultural waste products) is complicated and costly. In plants, ferulic acid is covalently bound to proteins and carbohydrates as a glycosidic conjugate, amide, or ester. Thus, the release of ferulic acid from plants requires chemical, enzymatic, or microbial hydrolysis. Despite the progress made in recent years in developing microbial methods for the extraction of ferulic acid from agro-industrial by-products, many advances are still required to render these methods efficient [57,157,158,159]. Chemical hydrolysis based on alkaline treatment at high temperatures reports a higher yield of ferulic acid than enzymatic hydrolysis. However, according to EU regulation, ferulic acid released by chemical processes cannot be considered a “natural” precursor. 

Today, ferulic acid used as a precursor for the commercial production of “natural vanillin” is mainly obtained from rice bran by enzymatic treatment. This recovery process is highly dependent on the availability of rice bran wastes and has high costs. Therefore, new “natural” ferulic acid sources might be considered to meet the global demand for this vanillin precursor. Some works have described the development of recombinant strains to produce ferulic acid from simple carbon sources [97,160,161]. Despite the low yields, this approach could be an exciting method to obtain cheap “natural” ferulic acid, which could be converted into bio-vanillin by vanillin-producing microorganisms after purification.

## 6. Conclusions

Vanillin is the most used flavouring agent in the food industry. “Natural vanillin” is highly demanded in the market, driving research to develop biotechnological approaches for bio-vanillin production. This review summarises and discusses the genetic engineering approaches to create recombinant strains for bio-vanillin production. This review also reports the considerable progress made in identifying and characterising key enzymes involved in vanillin biosynthesis. This knowledge presents an opportunity for the direct evolution of specific genes and the improvement of recombinant strains and fermentation conditions, leading to successful and low-cost industrial vanillin bioproduction.

## Figures and Tables

**Table 1:** Summary of vanillin production from different substrates using vanillin-producing or model microorganisms.

	Substrate	Strain	Figure/Section	Titre *	Yield ^#^	Reference
Vanillin-producing microorganisms	Ferulic acid	*Pseudomonas putida* KT2440 GN276	Figure 4 Section 4.2.1	0.73 g/L *	80%	[68]
		GN299		1.27 g/L *	83%	[68]
		GN442*∆areA*		1.25 g/L *	82%	[69]
		*Amycolatopsis* sp. ZYL926 WT		13.8 g/L	73%	[71]
		↑*fcs ech*		17.1 g/L	86%	
		*Amycolatopsis* sp. ATCC 39116 WT		12.2 g/L	69%	[70]
		*∆vdh*		14.4 g/L	81%	
		*∆vdh* ↑*fcs ech*		19.3 g/L	95%	
		*Amycolatopsis* sp. CCTCC No:M2011265 WT		7.25 g/L	60%	[74]
		*∆vdh*		9.13 g/L	76%	
		*∆vdh∆phdB*		9.88 g/L	82%	
	Lignin	*Rhodococcus jostii* RHA1 *∆vdh*	Figure 5 Section 4.2.1	96 mg/L	NC	[80]
		*Arthrobacter* sp. C2 *∆xylC* ↑*pchF*		57 mg/L	NC	
	Eugenol	*Amycolatopsis* sp. HR167 *+* [*vaoA*]	Figure 6	traces	NC	[82]
		*Streptomyces* sp. *+* [*vaoA*]	Section 4.2.2	-	30–72%	[83]
Model microorganisms	Isoeugenol	*E. coli* BL21 + [*iem]*	Figure 7 Sections Vanillin from Phenylpropanoids and Reduction of Vanillin Degradation	28.37 g/L	81%	[85]
	Eugenol	*E. coli* XL1-blue + [*vaoA*,*calA*,*calB*,*ech*,*fcs*]		0.3 g/L	8%	[87]
		*E. coli* BW25113 WT + [*vaoA*,*calA*,*ccr*,*ech*]		10.68 mg/L *	3.5%	[88]
		*E. coli* BW25113 Van-3 + [*vaoA*,*calA*,*ccr*,*ech*]		83.88 mg/L *	28%	
	Ferulic acid	*E. coli* JM109 construction A2	Figure 8 Section Vanillin from Phenylpropanoids	1.1 g/L	70%	[90]
		*E. coli* JM109 construction A3		0.36 g/L	78%	[91]
		*E. coli* JM109 construction A4 in fed-batch		4.26 g/L	88%	[92]
		*E. coli* BW25113 WT *+* [*ech*,*fcs*]	Section Optimisation of Metabolic Fluxes to Improve Cofactor and Precursor Supplies	0.79 g/L	34%	[121]
		*E. coli* BW25113 *∆icdA* ↑*gltA +* [*ech*,*fcs*]		2.21 g/L	92%	
		*E. coli* BL21 (DE3) *+* [*fdc*] and *E. coli* BL21 (DE3) *+* [*cso*] two stage process	Figure 11 Section De Novo Bioconversion Pathway from Ferulic Acid	7.93 g/L	69%	[104]
		*E. coli* BL21 (DE3) *+* [*ado*,*pad*]		4.22 g/L	92%	[105]
	Tyrosine	*E. coli* BL21 (DE3) *+* [*sam8*,*sam5*_,_*comt*,*fcs*,*ech*]	Figure 9 Section Vanillin from Aromatic Amino Acids	97.2 mg/L	5.8%	[94]
		*S. cerevisiae +* [*sam8*,*sam5*_,_*comt*,*fcs*,*ech*]		50.2 µg/L	NC	[96]
		aro4*aro7*+ [*sam8*,*sam5*_,_*comt*,*fcs*,*ech*]		700 µg/L	NC	
	Phenylalanine	*S. cerevisiae* YOOVAN	Figure 10 Section Vanillin from Aromatic Amino Acids	-	NC	[97]
		YOCPVAN		153 µg/L *	NC	[102]
	Glucose	*E. coli* KL7 construction Figure 13(A1) + in vitro aldehyde carboxylic acid reductase	Figure 12 and Figure 13 Section De Novo Bioconversion Pathways from Glucose and Section 4.3.3	2.76 g/L	16%	[107]
		*E. coli* RARE construction Figure 13(A2)		0.120 g/L	1.2%	[122]
		*E. coli* RARE *∆MetJ* construction Figure 13(A2)		0.256 g/L	2.5%	
		*S. pombe ∆ADH6* construction Figure 13(A3)		65 mg/L	0.4%	[109]
		*S. cerevisiae ∆ADH6* construction Figure 13(A3)		0.380 g/L	1.1%	[125]
		*Corynebacterium* construction Figure 13(A4)		0.31 g/L	0.9%	[111]
		*S. cerevisiae* JS-MARE3 VA1a		0.107 g/L	0.6%	[112]
		VA6		0.217 g/L	1.3%	
		VA8		0.262 g/L	1.5%	
		VA9		0.352 g/L	3.5%	
	Terephthalic acid	*E. coli RARE* construction Figure 14	Figure 14 Section De Novo Bioconversion Pathways from Glucose	119 mg/L	16%	[120]

* Vanillin titres are all presented in mass concentration to facilitate comparison and the conversion values are approximated to maximum of two decimal places; # Molar vanillin yields, NC—Not possible to calculate. Legend of symbols: ∆—gene deletion, ↑—overexpression of endogenous genes, […]—expression of exogenous genes, gene*—mutated genes (feedback resistant variants).

## Data Availability

Not applicable.

## Abbreviations

**Abbreviation**	**Description**, *gene*
**1,3DPG**	3-phospho-D-glyceroyl-phosphate
**3DSD**	3-dehydroshikimate dehydratase, *aroZ, 3DSD, asbF*
**4CL**	4-hydroxycinnamoyl-CoA ligase, *4CL*
**4HBS**	4-hydroxybenzaldehyde synthetase
**4PG**	4-n-propylguaiacol
**ACAR**	aldehyde carboxylic acid reductase, *car*
**Ac-CoA**	acetyl-coenzyme A
**Ace**	acetate
**AcP**	acetyl phosphate
**ADH**	alcohol dehydrogenase, *AHD6*
**ADO**	aromatic dioxygenase, *ado*
**AKR**	aldoketo reductases
**ALDR**	aldehyde reductases, *areA*
**APX1**	ascorbate oxidase
**ARO4**	DAHP synthase, *aro4*
**ARO7**	chorismate mutase, *aro7, csm*
**AroB**	DHQ synthase*, aroB*
**AroD**	DHQ dehydratase, *aroD*
**AroE**	Shikimate dehydrogenase, *aroE*
**AroF, AroG, AroH**	DAHP synthase feedback regulated isoenzymes, *aroF, aroG, aroH*
**BFD**	Benzoylformate decarboxylase, *BFD*
**C3H**	4-coumaric acid 3-hydroxylase, *sam5, hpaBC*
**C4H**	cinnamate-4-hydroxylase, *C4H*
**CADH**	coniferyl alcohol dehydrogenase, *calA*
**CALDH**	coniferyl aldehyde dehydrogenase, *calB*
**CCR**	cinnamoyl-CoA reductase, *ccr*
**COMT**	caffeic acid O-methyltransferase, *comt*
**CPL**	chorismate-pyruvate lyase, *ubiC*
**CPR**	cytochrome P450 reductase, *CPR*
**CSO**	carotenoid oxygenase, *cso2*
**DAHP**	3-deoxy-D-arabino-heptulosonate-7-phosphate
**DCDDH**	1,4-dicarboxylic acid dehydrogenase
**DHGA**	3,4-dihydroxyphenylglyoxylate
**DHMA**	3,4-dihydroxymandelate
**DHQ**	3-dehydroquinic acid
**DHS**	3-dehydroshikimic acid
**DyP**	dye-decolorising peroxidase
**E4P**	erythrose-4-phosphate
**ECH**	enoyl-CoA hydratase, *ech*
**EUGH**	eugenol hydroxylase, *ehyA, ehyB*
**F6P**	fructose 6-phosphate
**FCS**	feruloyl-CoA synthetase, *fcs*
**FDC**	ferulic acid decarboxilase, *fdc*
**G6P**	glucose 6-phosphate
**GAP**	D-glyceraldehyde 3-phosphate
**GapC**	GAP dehydrogenase
**GDH1**	NADP+ dependent glutamate dehydrogenases, *gdh1*
**GDH2**	NAD+ dependent glutamate dehydrogenases*, gdh2*
**GltA**	citrate synthase, *gltA*
**GPP1**	glycerol-1-phosphate, *gpp1*
**HBH**	Hydroxybenzoate hydroxylase, *pobA*, *hbh*
**HCHL**	4-hydroxycinnamate CoA-hydratase/lyase
**HCT**	hydroxycinnamoyl transferase
**HCys**	homocysteine
**HMA**	hydroxymandelate
**HmaS**	hydroxymandelate synthase, *HmaS*
**HMO**	hydroxymandelate oxidase, *HMO*
**HMPHP-CoA**	4-hydroxy-3-methoxyphenyl-β-hydroxypropionyl-CoA
**HpaBC**	Two-component flavin-dependent monooxygenase, *hpaBC*
**HPP**	hydroxyphenylpyruvate
**IcdA**	isocitrate dehydrogenase, *icdA*
**IEM**	isoeugenol monooxygenase, *iem*
**Lac**	laccase
**LiP**	lignin peroxidase
**LuxS**	S-ribosylhomocysteine lyase, *luxS*
**MARE**	minimal aromatic aldehyde reduction ability
**Met**	methionine
**MetE, MetH**	methionine synthases, *metE, metH*
**MetJ**	transcription regulator of methionine biosynthetic gene cluster, *metJ*
**MetK**	SAM synthetase, *metK*
**MnP**	manganese-dependent peroxidase
**Mtn**	5'-methylthioadenosine/S-adenosylhomocysteine nucleosidase, *mtn*
**OMT**	O-methyltransferase, *OMT, comt*
**PAD**	phenolic acid decarboxylase, *pad*
**PAL**	phenylalanine ammonia-lyase, *PAL*
**PCA**	protocatechuic acid
**PcaHG**	protocatechuate dioxygenase, *pcaHG*
**PDC1**	pyruvate decarboxylase
**PEP**	phosphoenolpyruvate
**PET**	polyethylene terephthalate
**PhdB**	HMPHP-CoA dehydrogenase, *phdB*
**Pos5c**	cytosol-relocalised NADH kinase, *pos5c*
**PPDO**	phenylpropanoid 2,3-dioxygenase
**PpsA**	phosphoenolpyruvate synthase, *ppsA*
**PPTase**	phophopantetheinyl transferase, *entD, sfp, PPT*
**Pta**	phosphotransacetylase, *pta*
**PtsH**	phosphocarrier protein, *ptsH*
**Pyr**	pyruvate
**RARE**	reduced aromatic aldehyde reduction ability
**ROS**	reactive oxygen species
**SAH**	S-adenosyl-L-homocysteine
**SAM**	S-adenosyl-L-methionine
**SerA**	phosphoglycerate dehydrogenase, *serA*
**SRH**	S-ribosyl-L-homocysteine
**TA**	terephthalic acid
**TAL**	ammonia-lyase activity, *sam8*
**TktA**	transketolase, *tktA*
**TPADO**	TA 1,2-dioxygenase, *tphA1, tphA2, tphB2*
**Trp**	tryptophan
**UDP**	uridine-diphophate activated sugar
**UGT**	UDP-glycosyltransferase
**VAO**	vanillyl alcohol oxidase, *vao*, *pchF*
**VDC**	vanillate decarboxylase, *vdcBCD*
**VDH**	vanillin dehydrogenase, *vdh*
**VDM**	vanillate demethylase, *vanAB*
**VpVAN**	vanillin synthase, *VpVan*
**VR**	vanillin reductase, *vr*
**Xfpk**	phosphoketolase, *xfpk*
**β-Gluc**	β-glucosidase

## References

[1] Spence C. (2022). Odour Hedonics and the Ubiquitous Appeal of Vanilla. Nat. Food.

[2] Martău G.A., Călinoiu L.F., Vodnar D.C. (2021). Bio-Vanillin: Towards a Sustainable Industrial Production. Trends Food Sci. Technol..

[3] Korthou H., Verpoorte R., Berger R.G. (2007). Vanilla. Flavours and Fragrances: Chemistry, Bioprocessing and Sustainability.

[4] GVR (2017). Vanillin Market Size, Share|Global Industry Report, 2018–2025.

[5] PMR (2020). Bio Vanillin Market.

[6] Gallage N.J., Møller B.L. (2015). Vanillin–Bioconversion and Bioengineering of the Most Popular Plant Flavor and Its De Novo Biosynthesis in the Vanilla Orchid. Mol. Plant.

[7] Dixon R.A., Havkin-Frenkel D., Belanger F.C. (2018). Vanillin Biosynthesis—Still Not as Simple as It Seems?. Handbook of Vanilla Science and Technology.

[8] Labuda I., Havkin-Frenkel D., Belanger F.C. (2018). Biotechnology of Vanillin: Vanillin from Microbial Sources. Handbook of Vanilla Science and Technology.

[9] Hérisson J., Duigou T., du Lac M., Bazi-Kabbaj K., Sabeti Azad M., Buldum G., Telle O., El Moubayed Y., Carbonell P., Swainston N. (2022). The Automated Galaxy-SynBioCAD Pipeline for Synthetic Biology Design and Engineering. Nat. Commun..

[10] Podstolski A., Havkin-Frenkel D., Belanger F.C. (2018). Enzymes Characterized From *Vanilla*. Handbook of Vanilla Science and Technology.

[11] Paul V., Rai D.C., Ramyaa R.L., Srivastava S.K., Tripathi A.D. (2021). A Comprehensive Review on Vanillin: Its Microbial Synthesis, Isolation and Recovery. Food Biotechnol..

[12] Liu Y., Sun L., Huo Y.-X., Guo S. (2023). Strategies for Improving the Production of Bio-Based Vanillin. Microb. Cell. Fact..

[13] Xu L., Liaqat F., Sun J., Khazi M.I., Xie R., Zhu D. (2024). Advances in the Vanillin Synthesis and Biotransformation: A Review. Renew. Sustain. Energy Rev..

[14] Gallage N.J., JÃ¸rgensen K., Janfelt C., Nielsen A.J.Z., Naake T., Duński E., Dalsten L., Grisoni M., MÃ¸ller B.L. (2018). The Intracellular Localization of the Vanillin Biosynthetic Machinery in Pods of *Vanilla planifolia*. Plant Cell Physiol..

[15] Havkin-Frenkel D., Belanger F.C. (2007). Application of Metabolic Engineering to Vanillin Biosynthetic Pathways in *Vanilla planifolia*. Applications of Plant Metabolic Engineering.

[16] Podstolski A., Havkin-Frenkel D., Malinowski J., Blounta J.W., Kourteva G., Dixon R.A. (2002). Unusual 4-Hydroxybenzaldehyde Synthase Activity from Tissue Cultures of the Vanilla Orchid *Vanilla planifolia*. Phytochemistry.

[17] Kundu A. (2017). Vanillin Biosynthetic Pathways in Plants. Planta.

[18] Fock-Bastide I., Palama T.L., Bory S., Lécolier A., Noirot M., Joët T. (2014). Expression Profiles of Key Phenylpropanoid Genes During Vanilla Planifolia Pod Development Reveal a Positive Correlation between PAL Gene Expression and Vanillin Biosynthesis. Plant Physiol. Biochem..

[19] Galadima A.I., Salleh M.M., Hussin H., Chong C.S., Yahya A., Mohamad S.E., Abd-Aziz S., Yusof N.N.M., Naser M.A., Al-Junid A.F.M. (2020). Biovanillin: Production Concepts and Prevention of Side Product Formation. Biomass Convers. Biorefinery.

[20] Gallage N.J., Hansen E.H., Kannangara R., Olsen C.E., Motawia M.S., Jørgensen K., Holme I., Hebelstrup K., Grisoni M., Møller B.L. (2014). Vanillin Formation from Ferulic Acid in *Vanilla planifolia* Is Catalysed by a Single Enzyme. Nat. Commun..

[21] Negishi O., Negishi Y. (2017). Phenylpropanoid 2,3-Dioxygenase Involved in the Cleavage of the Ferulic Acid Side Chain to Form Vanillin and Glyoxylic Acid in *Vanilla planifolia*. Biosci. Biotechnol. Biochem..

[22] Yang H., Barros-Rios J., Kourteva G., Rao X., Chen F., Shen H., Liu C., Podstolski A., Belanger F., Havkin-Frenkel D. (2017). A Re-Evaluation of the Final Step of Vanillin Biosynthesis in the Orchid *Vanilla planifolia*. Phytochemistry.

[23] Havkin-Frenkel D., Podstolski A., Dixon R.A. (2003). Vanillin Biosynthetic Pathway Enzyme from Vanilla planifolia.

[24] Chee M.J.Y., Lycett G.W., Khoo T.J., Chin C.F. (2017). Bioengineering of the Plant Culture of *Capsicum frutescens* with Vanillin Synthase Gene for the Production of Vanillin. Mol. Biotechnol..

[25] Arya S.S., Mahto B.K., Sengar M.S., Rookes J.E., Cahill D.M., Lenka S.K. (2022). Metabolic Engineering of Rice Cells with Vanillin Synthase Gene (VpVAN) to Produce Vanillin. Mol. Biotechnol..

[26] Nair R.B., Xia Q., Kartha C.J., Kurylo E., Hirji R.N., Datla R., Selvaraj G. (2002). Arabidopsis CYP98A3 Mediating Aromatic 3-Hydroxylation. Developmental Regulation of the Gene, and Expression in Yeast. Plant Physiol..

[27] Schoch G., Goepfert S., Morant M., Hehn A., Meyer D., Ullmann P., Werck-Reichhart D. (2001). CYP98A3 from *Arabidopsis thaliana* Is a 3′-Hydroxylase of Phenolic Esters, a Missing Link in the Phenylpropanoid Pathway. J. Biol. Chem..

[28] Franke R., Humphreys J.M., Hemm M.R., Denault J.W., Ruegger M.O., Cusumano J.C., Chapple C. (2002). The *Arabidopsis* REF8 Gene Encodes the 3-Hydroxylase of Phenylpropanoid Metabolism. Plant J..

[29] Barros J., Escamilla-Trevino L., Song L., Rao X., Serrani-Yarce J.C., Palacios M.D., Engle N., Choudhury F.K., Tschaplinski T.J., Venables B.J. (2019). 4-Coumarate 3-Hydroxylase in the Lignin Biosynthesis Pathway Is a Cytosolic Ascorbate Peroxidase. Nat. Commun..

[30] Pak F.E., Gropper S., Dai W.D., Havkin-Frenkel D., Belanger F.C. (2004). Characterization of a Multifunctional Methyltransferase from the Orchid *Vanilla planifolia*. Plant Cell Rep..

[31] Li H.M., Rotter D., Hartman T.G., Pak F.E., Havkin-Frenkel D., Belanger F.C. (2006). Evolution of Novel O-Methyltransferases from the *Vanilla planifolia* Caffeic Acid O-Methyltransferase. Plant Mol. Biol..

[32] Widiez T., Hartman T.G., Dudai N., Yan Q., Lawton M., Havkin-Frenkel D., Belanger F.C. (2011). Functional Characterization of Two New Members of the Caffeoyl CoA O-Methyltransferase-Like Gene Family from *Vanilla planifolia* Reveals a New Class of Plastid-Localized O-Methyltransferases. Plant Mol. Biol..

[33] Vogt T., Jones P. (2000). Glycosyltransferases in Plant Natural Product Synthesis: Characterization of a Supergene Family. Trends Plant Sci..

[34] Dignum M.J.W., van der Heijden R., Kerler J., Winkel C., Verpoorte R. (2004). Identification of Glucosides in Green Beans of *Vanilla planifolia* Andrews and Kinetics of Vanilla β-Glucosidase. Food Chem..

[35] Muheim A., Lerch K. (1999). Towards a High-Yield Bioconversion of Ferulic Acid to Vanillin. Appl. Microbiol. Biotechnol..

[36] Plaggenborg R., Overhage J., Loos A., Archer J.A.C., Lessard P., Sinskey A.J., Steinbüchel A., Priefert H. (2006). Potential of *Rhodococcus* Strains for Biotechnological Vanillin Production from Ferulic Acid and Eugenol. Appl. Microbiol. Biotechnol..

[37] Ghosh S., Sachan A., Sen S.K., Mitra A. (2007). Microbial Transformation of Ferulic Acid to Vanillic Acid by *Streptomyces sannanensis* MTCC 6637. J. Ind. Microbiol. Biotechnol..

[38] Plaggenborg R., Overhage J., Steinbüchel A., Priefert H. (2003). Functional Analyses of Genes Involved in the Metabolism of Ferulic Acid in *Pseudomonas putida* KT2440. Appl. Microbiol. Biotechnol..

[39] Zhao L.Q., Sun Z.H., Zheng P., Zhu L.L. (2005). Biotransformation of Isoeugenol to Vanillin by a Novel Strain of *Bacillus fusiformis*. Biotechnol. Lett..

[40] Yamada M., Okada Y., Yoshida T., Nagasawa T. (2007). Biotransformation of Isoeugenol to Vanillin by *Pseudomonas putida* IE27 Cells. Appl. Microbiol. Biotechnol..

[41] Chen P., Yan L., Wu Z., Li S., Bai Z., Yan X., Wang N., Liang N., Li H. (2016). A Microbial Transformation Using *Bacillus subtilis* B7-S to Produce Natural Vanillin from Ferulic Acid. Sci. Rep..

[42] Kotchaplai P., Ninrat J., Mahipant G., Vangnai A.S. (2022). Involvement of Cytochrome P450 in Organic-Solvent Tolerant *Bacillus subtilis* GRSW1-B1 in Vanillin Production via Ferulic Acid Metabolism. Fermentation.

[43] Singh A., Mukhopadhyay K., Ghosh Sachan S. (2019). Biotransformation of Eugenol to Vanillin by a Novel Strain *Bacillus safensis* SMS1003. Biocatal. Biotransformation.

[44] Zheng L., Zheng P., Sun Z., Bai Y., Wang J., Guo X. (2007). Production of Vanillin from Waste Residue of Rice Bran Oil by *Aspergillus niger* and *Pycnoporus cinnabarinus*. Bioresour. Technol..

[45] Mehmood T., Ahmed S., Waseem R., Saeed S., Ahmed W., Irfan M., Ullah A. (2022). Valorization of Fruit Peels into Biovanillin and Statistical Optimization of Process Using *Enterobacter hormaechei* Through Solid-State Fermentation. Fermentation.

[46] Tang P.L., Hassan O. (2020). Bioconversion of Ferulic Acid Attained from Pineapple Peels and Pineapple Crown Leaves into Vanillic Acid and Vanillin by *Aspergillus niger* I-1472. BMC Chem..

[47] Dos Santos Barbosa E., Perrone D., Do Amaral Vendramini A.L., Ferreira Leite S.G. (2008). Vanillin Production by Phanerochete *Chrysosporium* Grown on Green Coconut Agro-Industrial Husk in Solid State Fermentation. BioResources.

[48] Di Gioia D., Sciubba L., Ruzzi M., Setti L., Fava F. (2009). Production of Vanillin from Wheat Bran Hydrolyzates via Microbial Bioconversion. J. Chem. Technol. Biotechnol..

[49] Yamada M., Okada Y., Yoshida T., Nagasawa T. (2007). Purification, Characterization and Gene Cloning of Isoeugenol-Degrading Enzyme from *Pseudomonas putida* IE27. Arch. Microbiol..

[50] Ashengroph M., Nahvi I., Zarkesh-Esfahani H., Momenbeik F. (2011). Use of Growing Cells of *Pseudomonas aeruginosa* for Synthesis of the Natural Vanillin via Conversion of Isoeugenol. Iran. J. Pharm. Res..

[51] Ryu J.Y., Seo J., Unno T., Ahn J.H., Yan T., Sadowsky M.J., Hur H.G. (2010). Isoeugenol Monooxygenase and Its Putative Regulatory Gene Are Located in the Eugenol Metabolic Gene Cluster in *Pseudomonas nitroreducens* Jin1. Arch. Microbiol..

[52] Priefert H., Overhage J., Steinbüchel A. (1999). Identification and Molecular Characterization of the Eugenol Hydroxylase Genes (ehyA/ehyB) of *Pseudomonas* sp. Strain HR199. Arch. Microbiol..

[53] Fraaije M.W., Veeger C., Van Berkel W.J.H. (1995). Substrate Specificity of Flavin-Dependent Vanillyl-Alcohol Oxidase from *Penicillium simplicissimum* Evidence for the Production of 4-Hydroxycinnamyl Alcohols from 4-Allylphenols. Eur. J. Biochem..

[54] Overhage J., Priefert H., Rabenhorst J., Steinbüchel A. (1999). Biotransformation of Eugenol to Vanillin by a Mutant of *Pseudomonas* sp. Strain HR199 Constructed by Disruption of the Vanillin Dehydrogenase (Vdh) Gene. Appl. Microbiol. Biotechnol..

[55] Mitra A., Kitamura Y., Gasson M.J., Narbad A., Parr A.J., Payne J., Rhodes M.J.C., Sewter C., Walton N.J. (1999). 4-Hydroxycinnamoyl-CoA Hydratase/Lyase (HCHL)—An Enzyme of Phenylpropanoid Chain Cleavage from *Pseudomonas*. Arch. Biochem. Biophys..

[56] Janusz G., Pawlik A., Sulej J., Świderska-Burek U., Jarosz-Wilkołazka A., Paszczyński A. (2017). Lignin Degradation: Microorganisms, Enzymes Involved, Genomes Analysis and Evolution. FEMS Microbiol. Rev..

[57] Salvachúa D., Werner A.Z., Pardo I., Michalska M., Black B.A., Donohoe B.S., Haugen S.J., Katahira R., Notonier S., Ramirez K.J. (2020). Outer Membrane Vesicles Catabolize Lignin-Derived Aromatic Compounds in *Pseudomonas putida* KT2440. Proc. Natl. Acad. Sci. USA.

[58] Simon O., Klaiber I., Huber A., Pfannstiel J. (2014). Comprehensive Proteome Analysis of the Response of *Pseudomonas putida* KT2440 to the Flavor Compound Vanillin. J. Proteom..

[59] Jiménez J.I., Miñambres B., García J.L., Díaz E. (2002). Genomic Analysis of the Aromatic Catabolic Pathways from *Pseudomonas putida* KT2440. Environ. Microbiol..

[60] Meyer F., Pupkes H., Steinbüchel A. (2017). Development of an Improved System for the Generation of Knockout Mutants of *Amycolatopsis* sp. Strain ATCC 39116. Appl. Environ. Microbiol..

[61] Achterholt S., Priefert H., Steinbüchel A. (2000). Identification of *Amycolatopsis* sp. Strain HR167 Genes, Involved in the Bioconversion of Ferulic Acid to Vanillin. Appl. Microbiol. Biotechnol..

[62] Fleige C., Hansen G., Kroll J., Steinbüchel A. (2013). Investigation of the *Amycolatopsis* sp. Strain ATCC 39116 Vanillin Dehydrogenase and Its Impact on the Biotechnical Production of Vanillin. Appl. Environ. Microbiol..

[63] Singhvi M., Kim B.S. (2021). Lignin Valorization Using Biological Approach. Biotechnol. Appl. Biochem..

[64] Fleige C., Steinbüchel A. (2014). Construction of Expression Vectors for Metabolic Engineering of the Vanillin-Producing Actinomycete *Amycolatopsis* sp. ATCC 39116. Appl. Microbiol. Biotechnol..

[65] Meyer F., Netzer J., Meinert C., Voigt B., Riedel K., Steinbüchel A. (2018). A Proteomic Analysis of Ferulic Acid Metabolism in *Amycolatopsis* sp. ATCC 39116. Appl. Microbiol. Biotechnol..

[66] Contreras-Jácquez V., Rodríguez-González J., Mateos-Díaz J.C., Valenzuela-Soto E.M., Asaff-Torres A. (2020). Differential Activation of Ferulic Acid Catabolic Pathways of *Amycolatopsis* sp. ATCC 39116 in Submerged and Surface Cultures. Appl. Biochem. Biotechnol..

[67] Xu X., Liu Y., Du G., Ledesma-Amaro R., Liu L. (2020). Microbial Chassis Development for Natural Product Biosynthesis. Trends Biotechnol..

[68] Graf N., Altenbuchner J. (2014). Genetic Engineering of *Pseudomonas putida* KT2440 for Rapid and High-Yield Production of Vanillin from Ferulic Acid. Appl. Microbiol. Biotechnol..

[69] García-Hidalgo J., Brink D.P., Ravi K., Paul C.J., Lidénb G., Gorwa-Grauslund M.F. (2020). Vanillin Production in *Pseudomonas*: Whole-Genome Sequencing of *Pseudomonas* sp. Strain 9.1 and Reannotation of *Pseudomonas putida* CalA as a Vanillin Reductase. Appl. Environ. Microbiol..

[70] Fleige C., Meyer F., Steinbüchel A. (2016). Metabolic Engineering of the Actinomycete *Amycolatopsis* sp. Strain ATCC 39116 towards Enhanced Production of Natural Vanillin. Appl. Environ. Microbiol..

[71] Darricau M., Desfougères T., Pernodet J.-L. (2017). Bacterial Strains for the Production of Vanillin.

[72] Meng Y., Qiang S., Guo J., Niu Y., Yang L. (2023). Gene-Deficient Amycolatopsis Capable of Producing Vanillin at High Yield, and Construction Method and Application Thereof.

[73] Gelo-Pujic M., Amory A. (2016). Microorganisms and Methods for Producing Vanillin.

[74] Wang G., Zheng P., Wu D., Chen P. (2023). High-Yield Natural Vanillin Production by *Amycolatopsis* sp. after CRISPR-Cas12a-Mediated Gene Deletion. ACS Omega.

[75] Davis J.R., Goodwin L.A., Woyke T., Teshima H., Bruce D., Detter C., Tapia R., Han S., Han J., Pitluck S. (2012). Genome Sequence of *Amycolatopsis* sp. Strain ATCC 39116, a Plant Biomass-Degrading Actinomycete. J. Bacteriol..

[76] Zhou H., Xu Z., Cai C., Li J., Jin M. (2022). Deciphering the Metabolic Distribution of Vanillin in *Rhodococcus opacus* During Lignin Valorization. Bioresour. Technol..

[77] Xu Z., Peng B., Kitata R.B., Nicora C.D., Weitz K.K., Pu Y., Shi T., Cort J.R., Ragauskas A.J., Yang B. (2022). Understanding of Bacterial Lignin Extracellular Degradation Mechanisms by *Pseudomonas putida* KT2440 via Secretomic Analysis. Biotechnol. Biofuels Bioprod..

[78] McLeod M.P., Warren R.L., Hsiao W.W.L., Araki N., Myhre M., Fernandes C., Miyazawa D., Wong W., Lillquist A.L., Wang D. (2006). The Complete Genome of *Rhodococcus* sp. RHA1 Provides Insights into a Catabolic Powerhouse. Proc. Natl. Acad. Sci. USA.

[79] Chen H.P., Chow M., Liu C.C., Lau A., Liu J., Eltis L.D. (2012). Vanillin Catabolism in *Rhodococcus* jostii RHA1. Appl. Environ. Microbiol..

[80] Sainsbury P.D., Hardiman E.M., Ahmad M., Otani H., Seghezzi N., Eltis L.D., Bugg T.D.H. (2013). Breaking down Lignin to High-Value Chemicals: The Conversion of Lignocellulose to Vanillin in a Gene Deletion Mutant of *Rhodococcus jostii* RHA1. ACS Chem. Biol..

[81] Zhao X., Zhang Y., Jiang H., Zang H., Wang Y., Sun S., Li C. (2022). Efficient Vanillin Biosynthesis by Recombinant Lignin-Degrading Bacterium *Arthrobacter* sp. C2 and Its Environmental Profile via Life Cycle Assessment. Bioresour. Technol..

[82] Overhage J., Steinbüchel A., Priefert H. (2006). Harnessing Eugenol as a Substrate for Production of Aromatic Compounds with Recombinant Strains of *Amycolatopsis* sp. HR167. J. Biotechnol..

[83] Lambert F., Zucca J., Mane J. (2008). Method for Producing Aromatic Molecules in Streptomyces.

[84] Overhage J., Steinbüchel A., Priefert H. (2002). Biotransformation of Eugenol to Ferulic Acid by a Recombinant Strain of *Ralstonia eutropha* H16. Appl. Environ. Microbiol..

[85] Yamada M., Okada Y., Yoshida T., Nagasawa T. (2008). Vanillin Production Using *Escherichia coli* Cells Over-Expressing Isoeugenol Monooxygenase of *Pseudomonas putida*. Biotechnol. Lett..

[86] Zhao L., Xie Y., Chen L., Xu X., Zhao C.X., Cheng F. (2018). Efficient Biotransformation of Isoeugenol to Vanillin in Recombinant Strains of E*scherichia coli* by Using Engineered Isoeugenol Monooxygenase and Sol-Gel Chitosan Membrane. Process Biochem..

[87] Overhage J., Steinbüchel A., Priefert H. (2003). Highly Efficient Biotransformation of Eugenol to Ferulic Acid and Further Conversion to Vanillin in Recombinant Strains of *Escherichia coli*. Appl. Environ. Microbiol..

[88] Zhu X., Wu J., Li S., Xiang L., Jin J.-M., Liang C., Tang S.-Y. (2024). Artificial Biosynthetic Pathway for Efficient Synthesis of Vanillin, a Feruloyl-CoA-Derived Natural Product from Eugenol. J. Agric. Food Chem..

[89] Yoon S.H., Li C., Lee Y.M., Lee S.H., Kim S.H., Choi M.S., Seo W.T., Yang J.K., Kim J.Y., Kim S.W. (2005). Production of Vanillin from Ferulic Acid Using Recombinant Strains of *Escherichia coli*. Biotechnol. Bioprocess Eng..

[90] Yoon S.H., Li C., Kim J.E., Lee S.H., Yoon J.Y., Choi M.S., Seo W.T., Yang J.K., Kim J.Y., Kim S.W. (2005). Production of Vanillin by Metabolically Engineered *Escherichia coli*. Biotechnol. Lett..

[91] Barghini P., Di Gioia D., Fava F., Ruzzi M. (2007). Vanillin Production Using Metabolically Engineered *Escherichia coli* Under Non-Growing Conditions. Microb. Cell Factories.

[92] Luziatelli F., Brunetti L., Ficca A.G., Ruzzi M. (2019). Maximizing the Efficiency of Vanillin Production by Biocatalyst Enhancement and Process Optimization. Front. Bioeng. Biotechnol..

[93] Ye Q., Xu W., He Y., Li H., Zhao F., Zhang J., Song Y. (2023). Biosynthesis of Vanillin by Rational Design of Enoyl-CoA Hydratase/Lyase. Int. J. Mol. Sci..

[94] Ni J., Tao F., Du H., Xu P. (2015). Mimicking a Natural Pathway for de Novo Biosynthesis: Natural Vanillin Production from Accessible Carbon Sources. Sci. Rep..

[95] Generosa L., Wang S., Rokicki J., Ravindranath P., Hollingshead S., Yin D., Marshall A. iGEM 2007 Wiki Edinburgh/Self Flavouring Yoghurt. https://2007.igem.org/wiki/index.php/Edinburgh/Yoghurt/Modelling.

[96] Qiu D., Wang M., Zhou C., Zhao J., Zhang G. (2022). De Novo Biosynthesis of Vanillin in Engineered *Saccharomyces cerevisiae*. Chem. Eng. Sci..

[97] Ramaen O., Sauveplane V., Pandjaitan R. (2018). Recombinant Host Cell for Biosynthetic Production of Vanillin.

[98] Havkin-Frenkel D., Podstolski A. (2007). Vanillin Production.

[99] Verhoef S., Ruijssenaars H.J., de Bont J.A.M., Wery J. (2007). Bioproduction of P-Hydroxybenzoate from Renewable Feedstock by Solvent-Tolerant *Pseudomonas putida* S12. J. Biotechnol..

[100] Sutherland J.B., Crawford D.L., Pometto A.L. (1983). Metabolism of Cinnamic, p-Coumaric, and Ferulic Acids by *Streptomyces setonii*. Can. J. Microbiol..

[101] Sachan A., Ghosh S., Sen S.K., Mitra A. (2006). Co-Production of Caffeic Acid and p-Hydroxybenzoic Acid from p-Coumaric Acid by *Streptomyces caeruleus* MTCC 6638. Appl. Microbiol. Biotechnol..

[102] Ramaen O., Sauveplane V., Pandjaitan R., Gelo-Pujic M. (2015). Improved Production of Vanilloids by Fermentation.

[103] Furuya T., Miura M., Kino K. (2014). A Coenzyme-Independent Decarboxylase/Oxygenase Cascade for the Efficient Synthesis of Vanillin. Chembiochem A Eur. J. Chem. Biol..

[104] Furuya T., Miura M., Kuroiwa M., Kino K. (2015). High-Yield Production of Vanillin from Ferulic Acid by a Coenzyme-Independent Decarboxylase/Oxygenase Two-Stage Process. New Biotechnol..

[105] Ni J., Wu Y.-T., Tao F., Peng Y., Xu P. (2018). A Coenzyme-Free Biocatalyst for the Value-Added Utilization of Lignin-Derived Aromatics. J. Am. Chem. Soc..

[106] Fujimaki S., Sakamoto S., Shimada S., Kino K., Furuya T. (2024). Engineering a Coenzyme-Independent Dioxygenase for One-Step Production of Vanillin from Ferulic Acid. Appl Env. Microbiol.

[107] Li K., Frost J.W. (1998). Synthesis of Vanillin from Glucose. J. Am. Chem. Soc..

[108] Kunjapur A.M., Tarasova Y., Prather K.L.J. (2014). Synthesis and Accumulation of Aromatic Aldehydes in an Engineered Strain of *Escherichia coli*. J. Am. Chem. Soc..

[109] Hansen E.H., Møller B.L., Kock G.R., Bünner C.M., Kristensen C., Jensen O.R., Okkels F.T., Olsen C.E., Motawia M.S., Hansen J. (2009). De Novo Biosynthesis of Vanillin in Fission Yeast (S*chizosaccharomyces pombe*) and Baker’s Yeast (*Saccharomyces cerevisiae*). Appl. Environ. Microbiol..

[110] Hansen J., Hansen E.H., Sompalli H., Sheridan J., Heal J., Hamilton W. (2014). Compositions Ans Methods for the Biosynthesis of Vanillin or Vanillin Beta-D-Glucoside.

[111] Kim H.-S., Choi J.-A., Kim B.-Y., Ferrer L., Choi J.-M., Wendisch V.F., Lee J.-H. (2022). Engineered *Corynebacterium glutamicum* as the Platform for the Production of Aromatic Aldehydes. Front. Bioeng. Biotechnol..

[112] Mo Q., Yuan J. (2024). Minimal Aromatic Aldehyde Reduction (MARE) Yeast Platform for Engineering Vanillin Production. Biotechnol Biofuels.

[113] Hansen E.H., Hallwyl S.C., Hansen K. (2015). Compositions Ans Methods for the Biosynthesis of Vanillin or Vanillin Beta-D-Glucoside.

[114] Goldsmith N., Hansen E.H., Meyer J.-P., Brianza R. (2015). Methods of Improving Production of Vanillin.

[115] Liu L., Zhu Y., Chen Y., Chen H., Fan C., Mo Q., Yuan J. (2020). One-Pot Cascade Biotransformation for Efficient Synthesis of Benzyl Alcohol and Its Analogs. Chem. Asian J..

[116] Chen Y., Wu P., Ko L.-Y., Kao T.-Y., Liu L., Zhang Y., Yuan J. (2020). High-Yielding Protocatechuic Acid Synthesis from L-Tyrosine in *Escherichia coli*. ACS Sustain. Chem. Eng..

[117] Danso D., Chow J., Streita W.R. (2019). Plastics: Environmental and Biotechnological Perspectives on Microbial Degradation. Appl. Environ. Microbiol..

[118] Rolsky C., Kelkar V. (2021). Degradation of Polyvinyl Alcohol in US Wastewater Treatment Plants and Subsequent Nationwide Emission Estimate. Int. J. Environ. Res. Public Health.

[119] Mejía A.I., Lopez B.L., Hess M. (2003). Bioconversion of Poly(Vinyl Alcohol) to Vanillin in a Phanerochaete *Chrysosporum* Culture Medium. Mater. Res. Innov..

[120] Sadler J.C., Wallace S. (2021). Microbial Synthesis of Vanillin from Waste Poly(Ethylene Terephthalate). Green Chem..

[121] Lee E.G., Yoon S.H., Das A., Lee S.H., Li C., Kim J.Y., Choi M.S., Oh D.K., Kim S.W. (2009). Directing Vanillin Production from Ferulic Acid by Increased Acetyl-CoA Consumption in Recombinant *Escherichia coli*. Biotechnol. Bioeng..

[122] Kunjapur A.M., Hyun J.C., Prather K.L.J. (2016). Deregulation of S-Adenosylmethionine Biosynthesis and Regeneration Improves Methylation in the *E. coli* De Novo Vanillin Biosynthesis Pathway. Microb. Cell Factories.

[123] Brochado A.R., Matos C., Møller B.L., Hansen J., Mortensen U.H., Patil K.R. (2010). Improved Vanillin Production in Baker’s Yeast through In Silico Design. Microb. Cell Factories.

[124] Magasanik B., Kaiser C.A. (2002). Nitrogen Regulation in *Saccharomyces cerevisiae*. Gene.

[125] Brochado A.R., Patil K.R. (2013). Overexpression of O-Methyltransferase Leads to Improved Vanillin Production in Baker’s Yeast Only When Complemented with Model-Guided Network Engineering. Biotechnol. Bioeng..

[126] Jayakody L.N., Jin Y.S. (2021). In-Depth Understanding of Molecular Mechanisms of Aldehyde Toxicity to Engineer Robust *Saccharomyces cerevisiae*. Appl. Microbiol. Biotechnol..

[127] Iwaki A., Ohnuki S., Suga Y., Izawa S., Ohya Y. (2013). Vanillin Inhibits Translation and Induces Messenger Ribonucleoprotein (mRNP) Granule Formation in *Saccharomyces cerevisiae*: Application and Validation of High-Content, Image-Based Profiling. PLoS ONE.

[128] Wang X., Liang Z., Hou J., Bao X., Shen Y. (2016). Identification and Functional Evaluation of the Reductases and Dehydrogenases from *Saccharomyces cerevisiae* Involved in Vanillin Resistance. BMC Biotechnol..

[129] Hearn E.M., Patel D.R., Van Den Berg B. (2008). Outer-Membrane Transport of Aromatic Hydrocarbons as a First Step in Biodegradation. Proc. Natl. Acad. Sci. USA.

[130] Pattrick C.A., Webb J.P., Green J., Chaudhuri R.R., Collins M.O., Kelly D.J. (2019). Proteomic Profiling, Transcription Factor Modeling, and Genomics of Evolved Tolerant Strains Elucidate Mechanisms of Vanillin Toxicity in *Escherichia coli*. mSystems.

[131] Wang X., Liang Z., Hou J., Shen Y., Bao X. (2017). The Absence of the Transcription Factor Yrr1p, Identified from Comparative Genome Profiling, Increased Vanillin Tolerance Due to Enhancements of ABC Transporters Expressing, rRNA Processing and Ribosome Biogenesis in *Saccharomyces cerevisiae*. Front. Microbiol..

[132] Wada A., Prates É.T., Hirano R., Werner A.Z., Kamimura N., Jacobson D.A., Beckham G.T., Masai E. (2021). Characterization of Aromatic Acid/Proton Symporters in *Pseudomonas putida* KT2440 Toward Efficient Microbial Conversion of Lignin-Related Aromatics. Metab. Eng..

[133] Hua D., Ma C., Song L., Lin S., Zhang Z., Deng Z., Xu P. (2007). Enhanced Vanillin Production from Ferulic Acid Using Adsorbent Resin. Appl. Microbiol. Biotechnol..

[134] Ma X.K., Daugulis A.J. (2014). Transformation of Ferulic Acid to Vanillin Using a Fed-Batch Solid-Liquid Two-Phase Partitioning Bioreactor. Biotechnol. Prog..

[135] Li Z., Sun L., Wang Y., Liu B., Xin F. (2024). Construction of a Novel Vanillin-Induced Autoregulating Bidirectional Transport System in a Vanillin-Producing *E. coli* Cell Factory. J. Agric. Food Chem..

[136] Toth S., Lee K.J., Havkin-Frenkel D., Belanger F.C., Hartman T.G., Havkin-Frenkel D., Belanger F.C. (2018). Volatile Compounds in *Vanilla*. Handbook of Vanilla Science and Technology.

[137] Singh P., Khan S., Pandey S.S., Singh M., Banerjee S., Kitamura Y., Rahman L. (2015). ur Vanillin Production in Metabolically Engineered *Beta vulgaris* Hairy Roots Through Heterologous Expression of *Pseudomonas fluorescens* HCHL Gene. Ind. Crops Prod..

[138] Ni W., Zhang P., Long L., Ding S. (2022). Engineering and Linker-Mediated Co-Immobilization of Carotenoid Cleavage Oxygenase with Phenolic Acid Decarboxylase for Efficiently Converting Ferulic Acid into Vanillin. Process Biochem..

[139] Esparan V., Krings U., Struch M., Berger R. (2015). A Three-Enzyme-System to Degrade Curcumin to Natural Vanillin. Molecules.

[140] García-Bofill M., Sutton P.W., Guillén M., Álvaro G. (2019). Enzymatic Synthesis of Vanillin Catalysed by an Eugenol Oxidase. Appl. Catal. A Gen..

[141] Chimbazaza E., van der Sluis R., Marx S. (2022). Analyses of the Efficiency of a Recombinant Vanillyl Alcohol Oxidase in Producing Vanillin in the Hydrothermal Liquefaction Aqueous Phase. Biofuels.

[142] Liu H.M., Zou Y., Yao C.Y., Yang Z. (2020). Enzymatic Synthesis of Vanillin and Related Catalytic Mechanism. Flavour Fragr. J..

[143] Marić I., Guo Y., Fürst M.J.L.J., Van Aelst K., Van Den Bosch S., De Simone M., Martins L.O., Sels B.F., Fraaije M.W. (2023). A One-Pot, Whole-Cell Biocatalysis Approach for Vanillin Production Using Lignin Oil. Adv. Synth. Catal..

[144] Cai S.-J., Lin J.-C., Wang M.-Y., Ji X.-J., Zhang Z.-G. (2023). Biosynthesis of Vanillin from Vanillyl Alcohol by Recombinant *Escherichia coli* Cells Expressing 5-Hydroxymethylfurfural Oxidase. Ind. Crops Prod..

[145] Mostafa H.S., Hashem M.M. (2023). Lactic Acid Bacteria as a Tool for Biovanillin Production: A Review. Biotechnol. Bioeng..

[146] Song J., Lee E., Yoon S., Lee S., Lee J., Lee S., Kim S. Vanillin Production Enhanced by Substrate Channeling in Recombinant *E. coli*. Proceedings of the SIMB Annual Meeting and Exhibition. Industrial Microbiology and Biotechnology, Poster 125 (Session 1).

[147] Jang S., Gang H., Kim B.G., Choi K.Y. (2018). FCS and ECH Dependent Production of Phenolic Aldehyde and Melanin Pigment from L-Tyrosine in *Escherichia coli*. Enzym. Microb. Technol..

[148] Prieto M.A., Perez-Aranda A., Garcia J.L. (1993). Characterization of an *Escherichia coli* Aromatic Hydroxylase with a Broad Substrate Range. J. Bacteriol..

[149] Deng Y., Faivre B., Back O., Lombard M., Pecqueur L., Fontecave M. (2020). Structural and Functional Characterization of 4-hydroxyphenylacetate 3-hydroxylase from *Escherichia coli*. ChemBioChem.

[150] Furuya T., Arai Y., Kino K. (2012). Biotechnological Production of Caffeic Acid by Bacterial Cytochrome P450 CYP199A2. Appl. Environ. Microbiol..

[151] Dos Santos O.A.L., Gonçalves T.A., Sodré V., Vilela N., Tomazetto G., Squina F.M., Garcia W. (2022). Recombinant Expression, Purification and Characterization of an Active Bacterial Feruloyl-CoA Synthase with Potential for Application in Vanillin Production. Protein Expr. Purif..

[152] Seok J., Seo H., Hong J., Kim K.J. (2023). Production of Various Phenolic Aldehyde Compounds Using the 4CL-FCHL Biosynthesis Platform. Int. J. Biol. Macromol..

[153] Chen Q., Jiang Y., Kang Z., Cheng J., Xiong X., Hu C.Y., Meng Y. (2022). Engineering a Feruloyl–Coenzyme A Synthase for Bioconversion of Phenylpropanoid Acids into High-Value Aromatic Aldehydes. J. Agric. Food Chem..

[154] Sana B., Chia K.H.B., Raghavan S.S., Ramalingam B., Nagarajan N., Seayad J., Ghadessy F.J. (2017). Development of a Genetically Programed Vanillin-Sensing Bacterium for High-Throughput Screening of Lignin-Degrading Enzyme Libraries. Biotechnol. Biofuels.

[155] Kunjapur A.M., Prather K.L.J. (2019). Development of a Vanillate Biosensor for the Vanillin Biosynthesis Pathway in *E. coli*. ACS Synth. Biol..

[156] Zhao F., Zhang Y., Hu J., Shi C., Ao X., Wang S., Lin Y., Sun Z., Han S. (2023). Disruption of Phosphate Metabolism and Sterol Transport-Related Genes Conferring Yeast Resistance to Vanillin and Rapid Ethanol Production. Bioresour. Technol..

[157] Salvachúa D., Karp E.M., Nimlos C.T., Vardon D.R., Beckham G.T. (2015). Towards Lignin Consolidated Bioprocessing: Simultaneous Lignin Depolymerization and Product Generation by Bacteria. Green Chem..

[158] Reshmy R., Athiyaman Balakumaran P., Divakar K., Philip E., Madhavan A., Pugazhendhi A., Sirohi R., Binod P., Kumar Awasthi M., Sindhu R. (2022). Microbial Valorization of Lignin: Prospects and Challenges. Bioresour. Technol..

[159] Nirwana W.O.C., Hung I.H., Shu C.H. (2023). Enhancing the Bioconversion of Ferulic Acid from Alkaline Hydrolysate of Corn Cobs to Vanillin by Amycolatopsis Thermoflava Under Nutrient Limitation and Reducing Sugar Control. J. Chem. Technol. Biotechnol..

[160] Choi O., Wu C.Z., Kang S.Y., Ahn J.S., Uhm T.B., Hong Y.S. (2011). Biosynthesis of Plant-Specific Phenylpropanoids by Construction of an Artificial Biosynthetic Pathway in *Escherichia coli*. J. Ind. Microbiol. Biotechnol..

[161] Kang S.Y., Choi O., Lee J.K., Hwang B.Y., Uhm T.B., Hong Y.S. (2012). Artificial Biosynthesis of Phenylpropanoic Acids in a Tyrosine Overproducing *Escherichia coli* Strain. Microb. Cell Factories.

